# A computational model of stereoscopic prey capture in praying mantises

**DOI:** 10.1371/journal.pcbi.1009666

**Published:** 2022-05-19

**Authors:** James O’Keeffe, Sin Hui Yap, Ichasus Llamas-Cornejo, Vivek Nityananda, Jenny C. A. Read

**Affiliations:** 1 Dyson School of Design Engineering, Imperial College, London, United Kingdom; 2 Biosciences Institute, Newcastle University, Newcastle, United Kingdom; 3 School of Medical Education, Newcastle University, Johor, Malaysia; 4 Faculty of Psychology, Complutense University of Madrid, Madrid, Spain; The University of Edinburgh, UNITED KINGDOM

## Abstract

We present a simple model which can account for the stereoscopic sensitivity of praying mantis predatory strikes. The model consists of a single “disparity sensor”: a binocular neuron sensitive to stereoscopic disparity and thus to distance from the animal. The model is based closely on the known behavioural and neurophysiological properties of mantis stereopsis. The monocular inputs to the neuron reflect temporal change and are insensitive to contrast sign, making the sensor insensitive to interocular correlation. The monocular receptive fields have a excitatory centre and inhibitory surround, making them tuned to size. The disparity sensor combines inputs from the two eyes linearly, applies a threshold and then an exponent output nonlinearity. The activity of the sensor represents the model mantis’s instantaneous probability of striking. We integrate this over the stimulus duration to obtain the expected number of strikes in response to moving targets with different stereoscopic disparity, size and vertical disparity. We optimised the parameters of the model so as to bring its predictions into agreement with our empirical data on mean strike rate as a function of stimulus size and disparity. The model proves capable of reproducing the relatively broad tuning to size and narrow tuning to stereoscopic disparity seen in mantis striking behaviour. Although the model has only a single centre-surround receptive field in each eye, it displays qualitatively the same interaction between size and disparity as we observed in real mantids: the preferred size increases as simulated prey distance increases beyond the preferred distance. We show that this occurs because of a stereoscopic “false match” between the leading edge of the stimulus in one eye and its trailing edge in the other; further work will be required to find whether such false matches occur in real mantises. Importantly, the model also displays realistic responses to stimuli with vertical disparity and to pairs of identical stimuli offering a “ghost match”, despite not being fitted to these data. This is the first image-computable model of insect stereopsis, and reproduces key features of both neurophysiology and striking behaviour.

## Author summary

The praying mantis is the only insect so far known to compute depth using stereoscopic (3D) vision. Mantis stereopsis appears to be simpler than human stereopsis and most machine sterovision algorithms. A computational model of mantis stereopsis may therefore be beneficial to the field of robotics, particularly where computational power is limited. Using a combination of behavioural observations and neurophysiological data, we propose a very simple model structure to describe the prey capture response in the praying mantis. We used the limited available data on the mantis’ size and distance preferences for its prey to train our model parameters. Our simple model is able to qualitatively reproduce previously unexplained characteristics of our training data, and predicts key observations in additional empirical data that was not included in the model training. Whilst we believe our model to be only a partial and heavily simplified account of mantis stereopsis, our results are supportive of our model structure as an approximation of the size and disparity sensors used by the mantis when catching its prey.

## 1 Introduction

Depth estimation is a critical task for many natural organisms and has broad applicability for autonomous systems. Stereopsis, the computation of distance via triangulation between two eyes, stands out as a particularly robust solution for depth estimation. It has evolved independently at least five times: in mammals, birds, amphibians, cephalopods and insects [1–3].

Most machine stereopsis algorithms tend to draw inspiration from humans: comparing patterns of contrast in the two eyes’ images to calculate a detailed map of disparity across the visual field [4, 5]. This requires the solution of the stereoscopic correspondence problem, i.e. figuring out which point in the left eye is viewing the same point in space as a given point in the right eye. It is a computationally expensive process, requiring considerable processing power. In humans, it appears to involve multiple areas of visual cortex [6], and in machines the performance of such algorithms is strongly restricted by the availability of computational resources [7]. This limits their applicability to areas such as swarm robotics, where individual robots are necessarily cheap and lightweight [8].

**Fig 1:**
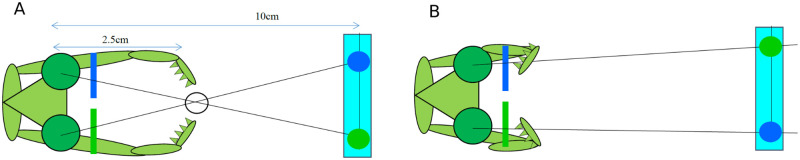
Praying mantis viewing a simulated target in (A) crossed and (B) uncrossed geometry. Each element of the target is displayed on a screen 10 cm away, well outside the catch range. Coloured filters placed over the mantid’s eyes ensure that each eye sees only the intended target. In (A), the target is simulated as being at 2.5 cm, where the lines of sight cross, eliciting a strike. In (B), left and right images are exchanged so the lines of sight diverge. mantids rarely strike at such stimuli [10, 11].

One way of addressing this limitation would be to draw inspiration from stereo algorithms in animals with smaller brains relative to primates. Insects are particularly interesting animals in which to investigate this given that their brains are orders of magnitude smaller than human brains. Despite this they are capable of complex visual tasks. Praying mantids are the only insects so far proven to use stereopsis for depth estimation [9–11]. Experiments have demonstrated this by modifying the disparity between mantis eyes with prisms [9] and more recently, an “insect 3D cinema” using coloured filters to display separate images to each eye (Fig 1). In this paper we therefore develop a basic model of mantis stereopsis.

### 1.1 Mantis vs human stereopsis

One way in which mantis stereopsis seems simpler than humans’ is that it probably aims to compute only whether or not a prey item is within catch range, rather than to compute a map of disparity across the visual scene [12]. Additionally, our behavioural experiments have revealed that mantids compute stereoscopic distance using stimulus features different to those used by human stereopsis [13]. Humans do have some weak ability to use “kinetic disparity”, i.e. disparity defined by temporal change [14, 15]. However, our normal stereoscopic perception relies overwhelmingly on comparing the detailed pattern of contrast in the two eyes. One demonstration of this is the profound disruption of human stereopsis produced by inverting contrast polarity in one eye, so that a bright feature in one eye now corresponds to a dark feature in the other [16–20].

In contrast, mantis stereopsis is not at all sensitive to the detailed pattern of contrast, but seems to depend solely on kinetic disparity between regions of the image where contrast is changing. For example, mantis stereopsis is quite happy to accept as “matches” between left and right eyes regions where the contrast is opposite in sign (i.e. light vs dark) or where local elementary motion is opposite in direction (e.g. up vs down) [13, 21]. This suggests that the inputs to mantis stereopsis are quite different from the inputs to vertebrate stereopsis, e.g. with temporal filtering plus rectification to obtain sensitivity to change but insensitivity to contrast polarity.

**Fig 2:**
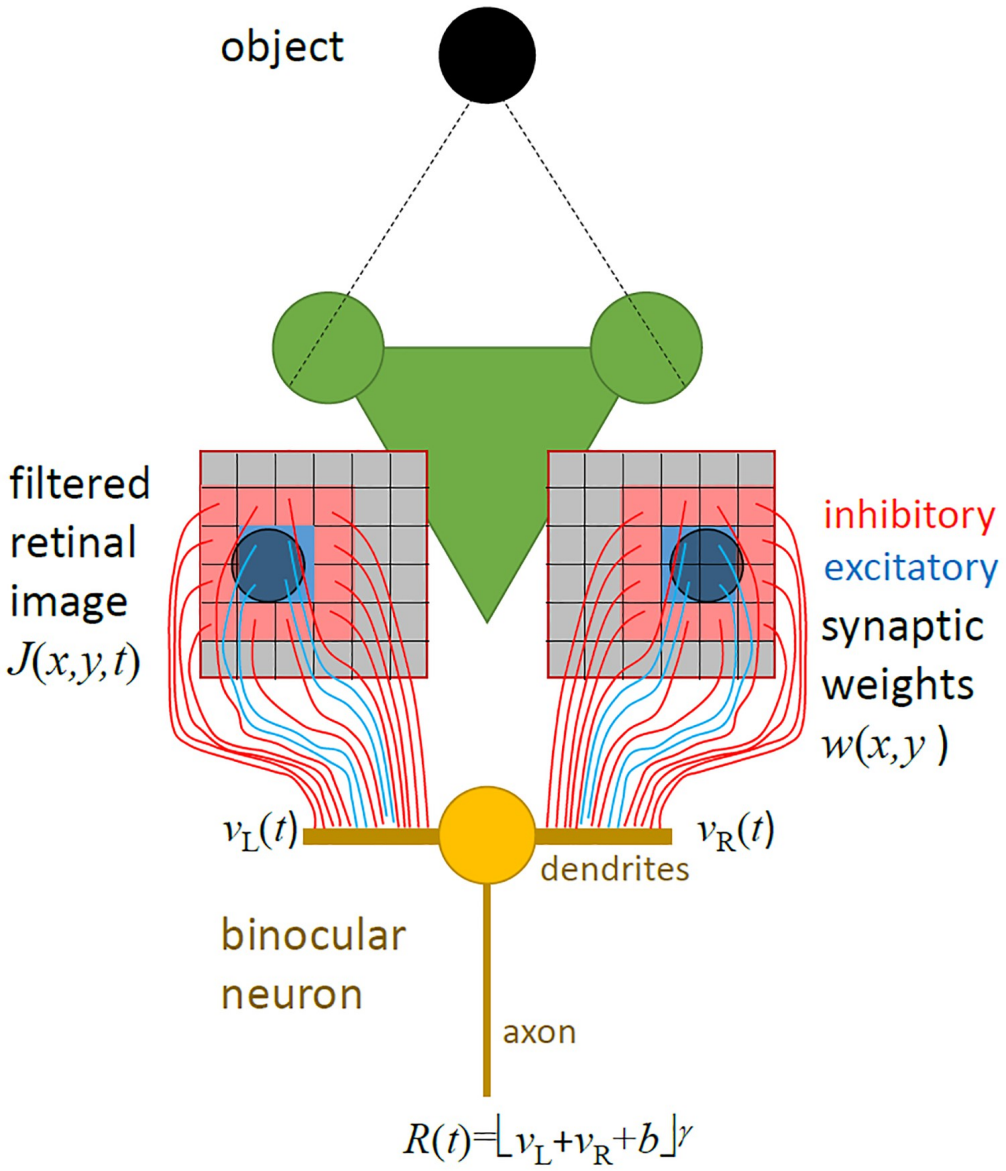
Schematic diagram of the disparity energy model. The model postulates a binocular neuron which receives input from each eye, representing the inner product of the retinal image with a receptive field function. The receptive field function represents the effect of stimulation at each point in the retinal image on the binocular neuron, and can be thought of as an effective synaptic weight (though the real pathway is multisynaptic). Red and blue are used to represent inhibitory and excitatory weights respectively. The activity of the neuron is then represented as a nonlinear function of its total input. In the original energy model [24], this nonlinearity was a threshold at 0 followed by squaring; we generalise the model to allow for a non-zero threshold −*b* and arbitrary exponent *γ*. Mathematical symbols are defined below, Eqs (1) and (2). We postulate that this binocular neuron synapses onto a motoneuron in such a way that the instantaneous strike probability is proportional to the activity of the binocular neuron.

Intriguingly, however, neurophysiological experiments suggest that the basic computation performed on these inputs by disparity-selective neurons in the mantis brain may be quite similar in insects and vertebrates [2, 22, 23]. A slightly modified version of the disparity energy model, originally introduced to explain the response of neurons in primary visual cortex of the cat [24] and later applied to primates [16, 25, 26], gave a good account of most neurons recorded in the mantis brain [22, 23]. As shown in Fig 2, the energy model postulates a binocular neuron with linear receptive fields in each eye. The activity of the neuron is modelled as depending on the total input from the two eyes, after this has gone through an output nonlinearity (thresholding and squaring, in the original energy model). This model is also structurally very similar to that proposed by Kral and Prete [27], although in their proposal information processing within the brain is entirely monocular, with binocular information combined only at the level of a thoracic motor neuron.

**Fig 3:**
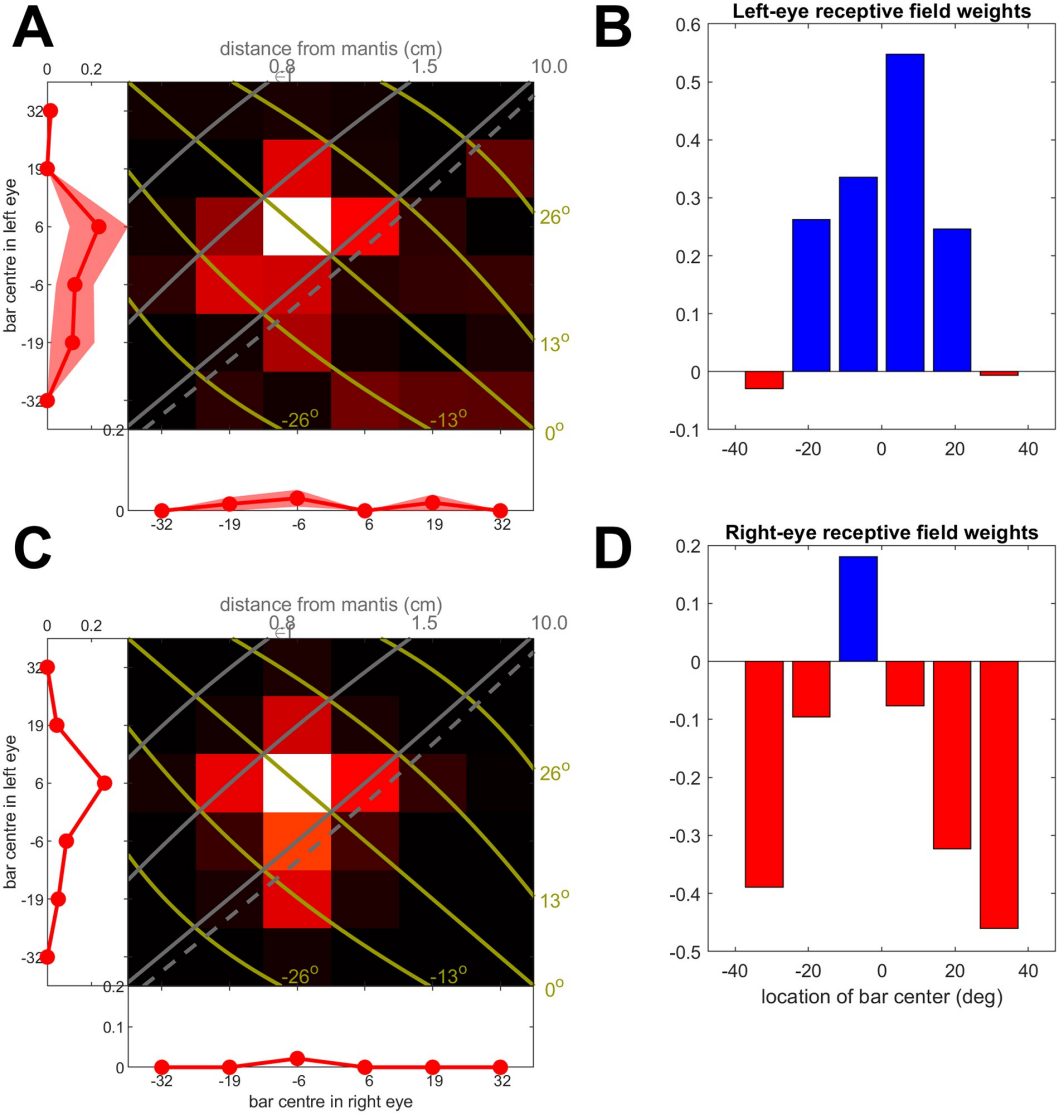
Response of real neuron (A, columnar commissural neuron rr160127 from [22]), along with response (C) predicted by the disparity energy model with the receptive field functions shown in B,D for left, right eyes. Here, due to experimental constraints on recording time, the stimuli were long vertical bars 13° in width, so the receptive fields are obtained only coarsely and as a function of only horizontal position. The receptive fields in both eyes have an excitatory centre (positive weights, blue) and inhibitory surround (negative, red). In the receptive fields fitted to this particular neuron, the excitatory centre is wider in the left eye than the right eye; elsewhere in this paper, we enforced symmetry for simplicity. The fitted threshold here is *b* = 0 and the output exponent is *γ* = 2.24; see Eqs (1) and (2). See [22] and its Supplementary Information for a detailed description of how responses are plotted in A and C for the real and model neuron respectively. Briefly, the marginal plots show the responses to monocular bars flashed in left or right eyes. The pseudocolor shows the responses to binocular bars, with the location in left, right eyes given by the location along vertical, horizontal axes. The olive lines mark the azimuthal location of the stimulus and the gray lines its disparity (dashed line indicates infinite simulated distance, or zero disparity). This neuron responded best to a bar flashed at a location corresponding to 2.5cm away on the midline.

Fig 3 shows an example of how well this very simple model structure is able to account for currently available data on mantis neurophysiology. Fig 3A shows the responses of a columnar commissural mantis neuron from [22] to monocular (marginal plots) and binocular (pseudocolor) stimuli of diameter 13° at varying stereoscopic disparities and azimuths (grey, olive contour lines). Fig 3C shows the responses of an energy-model neuron with the receptive field functions shown in Fig 3B and 3D. The fitted model agrees closely with the data. This suggests that mammals and mantids may have independently involved the same basic binocular computation, closely related to cross-correlation [28, 29], although the inputs to this computation may be different: contrast in vertebrates vs temporal change in mantids.

Overall, then, it seems that mantis stereopsis has evolved some of the same techniques as human stereopsis, but uses them within a simpler and computationally cheaper system, while achieving results that have made the praying mantis a successful ambush predator for millions of years.

### 1.2 Sensitivity both to target size and stereoscopic disparity

As in the example in Fig 3, many mantis binocular neurons have receptive fields with an excitatory center flanked by an inhibitory surround [22, 23]. This was an exciting finding because Rossel [30] had previously postulated binocular neurons with just this receptive field structure while discussing how mantids solve the stereo correspondence problem, and specifically how they avoid erroneous responses to “ghost matches”, created where lines of sight to two identical, distant objects intersect so as to simulate a virtual object at the intersection point. Rossel had previously suggested that a similar scheme might account for mantis responses to stimuli with vertical disparity [31].

This binocular receptive field structure naturally makes a neuron sensitive to both size and disparity. With regard to size tuning, the preferred size corresponds to the central excitatory region of the receptive field. Smaller prey would not fill this excitatory region, and would therefore not maximally activate the neuron, while larger prey would also activate the inhibitory surround, reducing the response. The disparity tuning would arises from the relative position of the receptive fields between the two eyes [24] (cf examples in Fig 3B and 3D, where the receptive field function peaks at +6° in the left eye but at −6° in the right eye).

As well as being sensitive to stereoscopically-defined distance, mantids are also most likely to attack prey of a particular angular size, typically 10-30° [32–36]. That is, mantis striking behaviour is sensitive to both size and disparity. Our first question therefore is how the size and disparity tuning of mantis behavior relates to the size and disparity tuning of individual neurons.

### 1.3 A single-sensor model of praying mantis striking behavior

In principle, the dependence of mantis striking behaviour on size and disparity could reflect the activity of distinct neurons: some neuron(s) tuned to size but not disparity, and others tuned to disparity but not size, both influencing strike probability. As noted, we also know from neurophysiology data that neurons tuned to both size and disparity exist in the mantis brain. In fact, mantis brains contain multiple distinct classes of such neurons [22, 23], and it is likely that mantis behavior reflects activity in many such neurons. Even if only a single neuron class were involved in striking behaviour, strikes might reflect activity in several different neurons of this class, e.g. tuned to different locations in space; neuromimetic models of human stereopsis, e.g. [28], invariably assume a population of neurons tuned to different disparities.

However, in this first paper we chose to ignore such complexities and examine just a single disparity-tuned model neuron or “disparity sensor”, tuned to a single location in space. Establishing the strengths and limitations of a single-neuron model is an essential prerequisite for understanding the additional contributions of other classes of neuron, as well as for discovering the simplest model that can achieve stereopsis. We therefore examined how well this simple model can capture key aspects of mantis predatory behaviour, including its tuning to prey size and vertical disparity as well as stereoscopic distance or horizontal disparity. We had two specific questions about size and disparity tuning which we expected would challenge a single-sensor model.

While we were confident that a single neuron could be made sensitive both to size and disparity, qualitatively in agreement with mantis behaviour, we were less confident that it could account quantitatively for the data. Rossel [36], using the species *Sphodromantis viridis*, found that mantis strike rate peaked for targets around 25° in diameter, falling to 50% maximal for targets as small as 15° or as large as 45°. Thus, the full-width half-maximum bandwidth of size tuning was around 30°. In a slightly smaller species, *Sphodromantis lineola*, we [10] found strike rates 50% of maximum for targets from 7 to 25°, i.e. a wide size-tuning bandwidth of around 18°. In contrast, both groups found much narrower disparity tuning. Rossel found that the peak strike rate dropped from 1 for 35mm to 0.6 for stimuli at 55mm, a change of 5° in binocular disparity given the interocular distance of 8mm. We [10] found similar sensitivity, with the strike rate dropping from 80% at 25mm to 20% at 38mm: also a change of around 5° in disparity, given the 7mm interocular distance of our mantids. Thus, the full width half-maximum bandwidth of disparity tuning corresponds to around 10° disparity, or an average shift of 5° in each eye.

**Fig 4:**
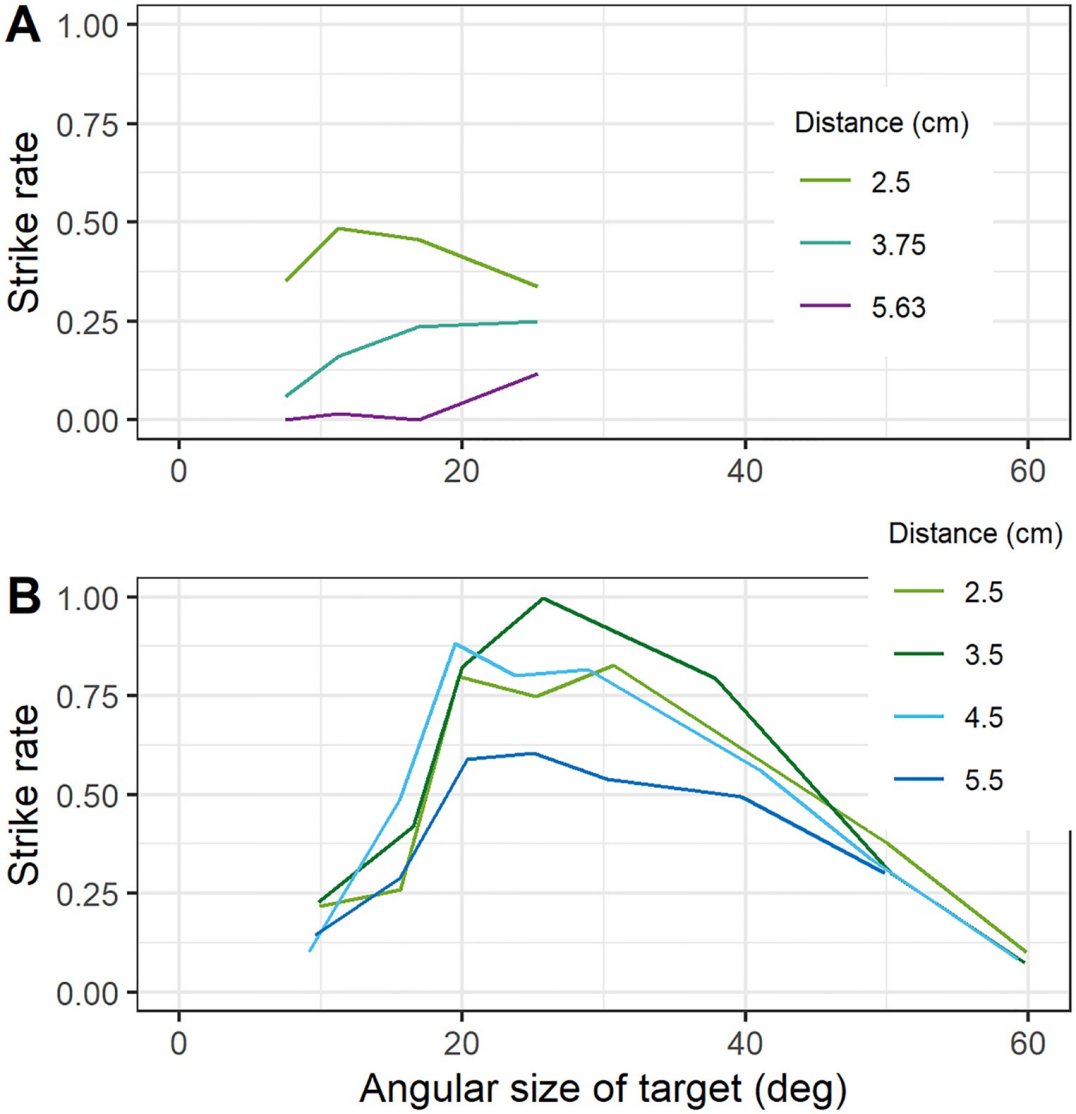
Sensitivity of mantis striking behaviour to stimulus size and stereoscopic distance. (A) Data from [10], available at http://dx.doi.org/10.1098/rstb.2015.0262. Species was *Sphodromantis lineola*; stimuli were on a computer screen 10cm from the insect; nearer distances were simulated using coloured filters. (B) Data extracted from Fig 5 of [36] using WebPlotDigitiser (https://apps.automeris.io/wpd/). Species was *Sphodromantis viridis*; stimuli were on a computer screen 5.5cm from the insect; nearer distances were simulated using base-out prisms. Note that in this figure, we plot the strike rate, i.e. the probability that a given trial elicits striking behaviour (since this was what was available for [36]). In [10], some trials elicited several strikes. The peak strike rate in A, 0.49, is lower than the peak of the mean number of strikes, plotted below in Fig 9.

We therefore wanted to find out whether it is possible to build a sensory neuron whose response stays high over a ∼20° range of target size, but tolerates only a 5° shift in monocular position, as indicated by the data in Fig 4. We thought this would be potentially challenging to achieve, since a neuron tuned to large sizes would necessarily have a large excitatory region and thus be tolerant of small monocular shifts which kept the target largely within this region. If indeed it was not possible to achieve this with a single sensor, this would suggest that mantis striking behaviour might be driven by a combination of several disparity sensors with small receptive fields. The behavioral disparity sensitivity would reflect the receptive field size of individual disparity sensors, while the size preference would reflect pooling of several such sensors with an excitatory-centre/inhibitory-surround pattern of weights. This would be important to know when attempting to unravel the underlying neural circuitry.

A second question of interest concerns the relationship between disparity and preferred angular size. Size constancy, i.e. a preference for prey of a constant physical size (in cm) regardless of distance, would require a decrease in preferred angular size (in degrees) as the distance increases. Previous work has found no evidence for size constancy [10, 36]. However, while Rossel [36] found that mantids preferred prey of around 25° regardless of distance, we [10] found that preferred angular size actually increased with stereoscopic disparity distance (Fig 4)—the opposite of what would be required for size constancy. It was unclear from previous results why this pattern emerged and the mechanisms underlying it are still unknown. Thus, a second aim of this paper was to understand whether this effect implies the operation of distinct disparity sensors, e.g. one tuned to a stereoscopic distance of 25mm and size 11°, and a second tuned to a distance of 40mm and size 25°. If so, this could provide one possible function for the different classes of disparity-tuned mantis neuron identified by Rosner [22, 23].

To address this, we constructed a model based on a single neuron tuned to both size and disparity, and sought model parameters that could account for our previously published behavioral data [10]. We found that a single-neuron model was able to account for the data surprisingly well. It could produce high strike rates over a large range of target sizes, while remaining sensitive to small changes in disparity (and thus even smaller changes in monocular position). To our surprise, this single sensor was also able to shift its size tuning so as to prefer larger angular sizes at larger stereoscopic disparities, as observed in mantids. We then tested the fitted model on independent data-sets using highly artificial stimuli with vertical disparity and with ghost matches. These data-sets were not used to fit the model parameters. We found that without any changes to parameters, the model produced qualitatively correct responses. The predicted strike rate decreased as vertical disparity increased, falling to zero for vertical disparities of around 15°, independent of the size of the stimulus, as observed empirically. And the strike rate was also substantially reduced when the virtual target is a “ghost match”, i.e. a local match that is not consistent with a global solution of the stereo correspondence problem. Most stereo algorithms achieve this via excitatory/inhibitory connections between disparity sensors, but the very limited scope of mantis stereopsis means that similar behavior can be achieved with just a single sensor. We conclude that this very simple model already gives a good account of much of the known data on mantis stereoscopic striking behaviour.

## 2 Methods

We first describe our model at a conceptual level (see Fig 2), before detailing how the simulation was implemented in Matlab.

### 2.1 Model structure

**Fig 5:**
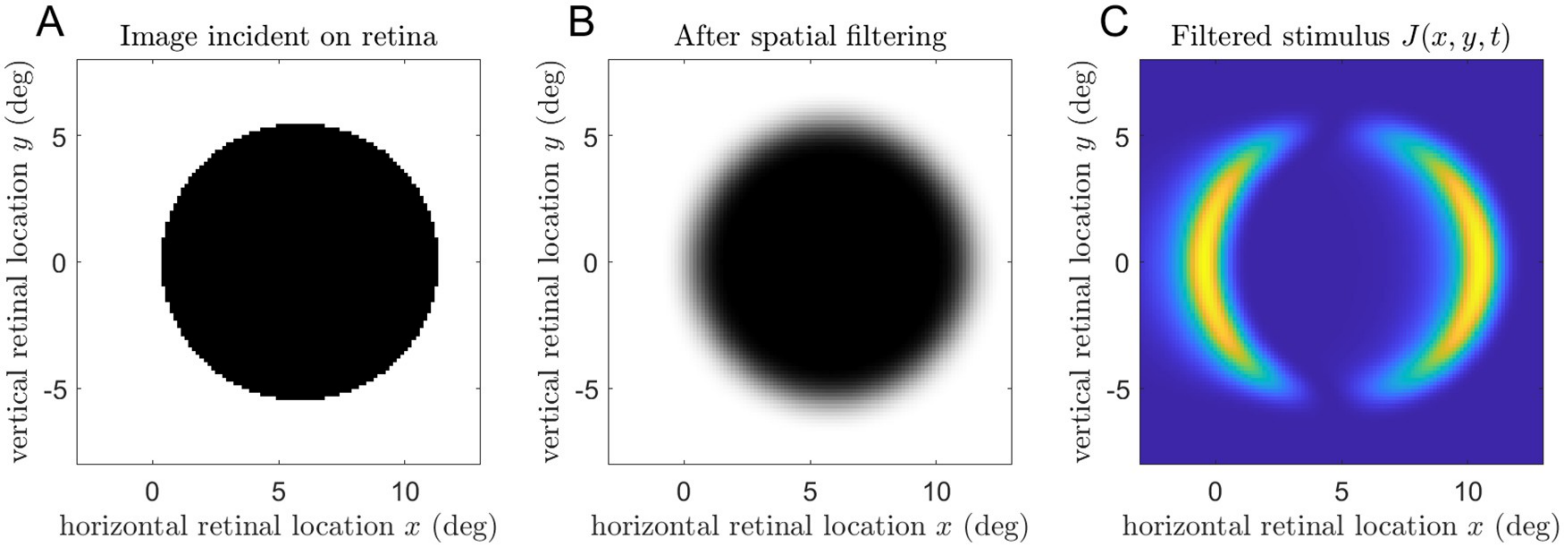
Spatiotemporal filtering, for a disk of diameter 11° moving from left to right. A: Pattern of light incident on the retina at a particular moment of time. B: After spatial filtering through a Gaussian with a standard deviation of 0.7°, representing the acceptance angle of foveal mantis ommatidia. C: The resulting neural input, after temporal filtering and squaring. There is activity only at the leading and trailing edges of the disk, where the retinal stimulus is changing.

Following our previous work [21], we assume that the images presented to the mantid’s eye first undergo lowpass spatial filtering, representing the response of retinal ommatidia to the pattern of light falling on the eye. They then undergo highpass temporal filtering, representing the response of lamina monopolar cells, which respond mainly to light increments and decrements [37, 38]. Following [39], we used a first-order Butterworth filter with a time-constant of 20ms. We then square the outputs, effectively combining responses to increments and decrements while losing the sign. We denote the result by *J*(*x*, *y*, *t*), a function of location on the retina and of time. This process is illustrated in Fig 5. All the symbols used in this paper and their meanings are listed in Table 1.

**Table 1:** Symbols used in the paper. Symbols used for model parameters are given separately in Table 2, along with fitted values.

Symbol	Meaning	Value or reference
*α*	Screen disparity, i.e. the angle subtended by the screen parallax at the point midway between the mantis’ eyes.	
*D*	Simulated stereoscopic distance of a stimulus from the mantis’ eyes (cm).	
Δ	Retinal disparity, i.e. the angular difference between a stimulus’ location in left and right eyes, assuming that 0° is directly ahead.	
*I*	Interocular separation of the mantis.	7mm
*J*(*x*, *y*, *t*)	Retinal image after low-pass spatial filtering, high-pass temporal filtering and squaring.	
$M_{data}^{j}$	Mean number of strikes empirically elicited per trial in stimulus condition *j*, averaged across all mantids	
$M_{model}^{j}\left(p\right)$	Mean number of strikes which the model predicts will be elicited per trial in stimulus condition *j*	
$N_{trials}^{j}$	Number of trials carried out for stimulus condition *j*	68
**p**	Complete set of model parameters	See Table 2
*P*	Screen parallax, i.e. physical separation of the left and right eye images on the stimulus display screen (cm).	
*R*(*t*)	Instantaneous response of the binocular neuron at time *t*	
*s*	Target diameter (deg).	
*S*	Physical distance of the stimulus display screen from the mantis’ eyes.	10cm
*V*_*tgt*_	Velocity of the moving target.	82°/*s*
*v*_*L*,*R*_(*t*)	Input to the binocular neuron from left, right eye	
*w*_*L*,*R*_(*x*, *y*)	Receptive field function in left, right eye; effectively, the synaptic weight connecting input from location (*x*, *y*) to the binocular neuron.	

In [22], we found that the response of disparity-tuned neurons in the mantis brain was well described with a model based on the disparity energy model originally proposed by [24]. In this model, images are convolved with linear receptive fields in each eye. The output from each receptive field is

$$\begin{array}{c} v(t)=\int dxdyJ(x,y,t)w(x,y) \end{array}$$ (1)

where *J*(*x*, *y*, *t*) is the filtered input from that eye, as a function of location and time, and *w*(*x*, *y*) is the receptive field function—effectively, the synaptic weight connecting input from location (*x*, *y*) to the disparity-tuned neuron. The instantaneous response of the disparity-tuned neuron is then modelled as

$$\begin{array}{c} R\left(t\right)={\left\lfloor v_{L}\left(t\right)+v_{R}\left(t\right)+b\right\rfloor }^{\gamma } \end{array}$$ (2)

In our fitted model, the bias *b* is negative and so acts as a threshold: the input *v*_*L*_ + *v*_*R*_ must exceed *b* in order for the response *R* to be non-zero. A positive value of *b* would model a tonic response, i.e. the cell would be active spontaneously even in the absence of input. The exponent *γ* controls the response selectivity. For example, suppose that with *γ* = 1, the optimal stimulus elicits a response of 1 unit while a less-preferred stimulus elicits a response of only 0.25 units, i.e. four times lower. If we now make *γ* = 2, the less-preferred stimulus now elicits a response of 0.0625, i.e. 16 times less than the optimal response.

We assume that the response of this neuron represents the instantaneous probability that the mantis will strike. Thus, the mean number of strikes released to a particular stimulus is obtained by integrating *R*(*t*) over the duration of the stimulus:

$$\begin{array}{c} M_{model}=\int_{stimulus}dtR\left(t\right) \end{array}$$ (3)

As described below, we adjust the model parameters so as to ensure good agreement between this expected number of strikes, predicted by the model, and the number of strikes observed empirically.

### 2.2 Receptive field structure

**Fig 6:**
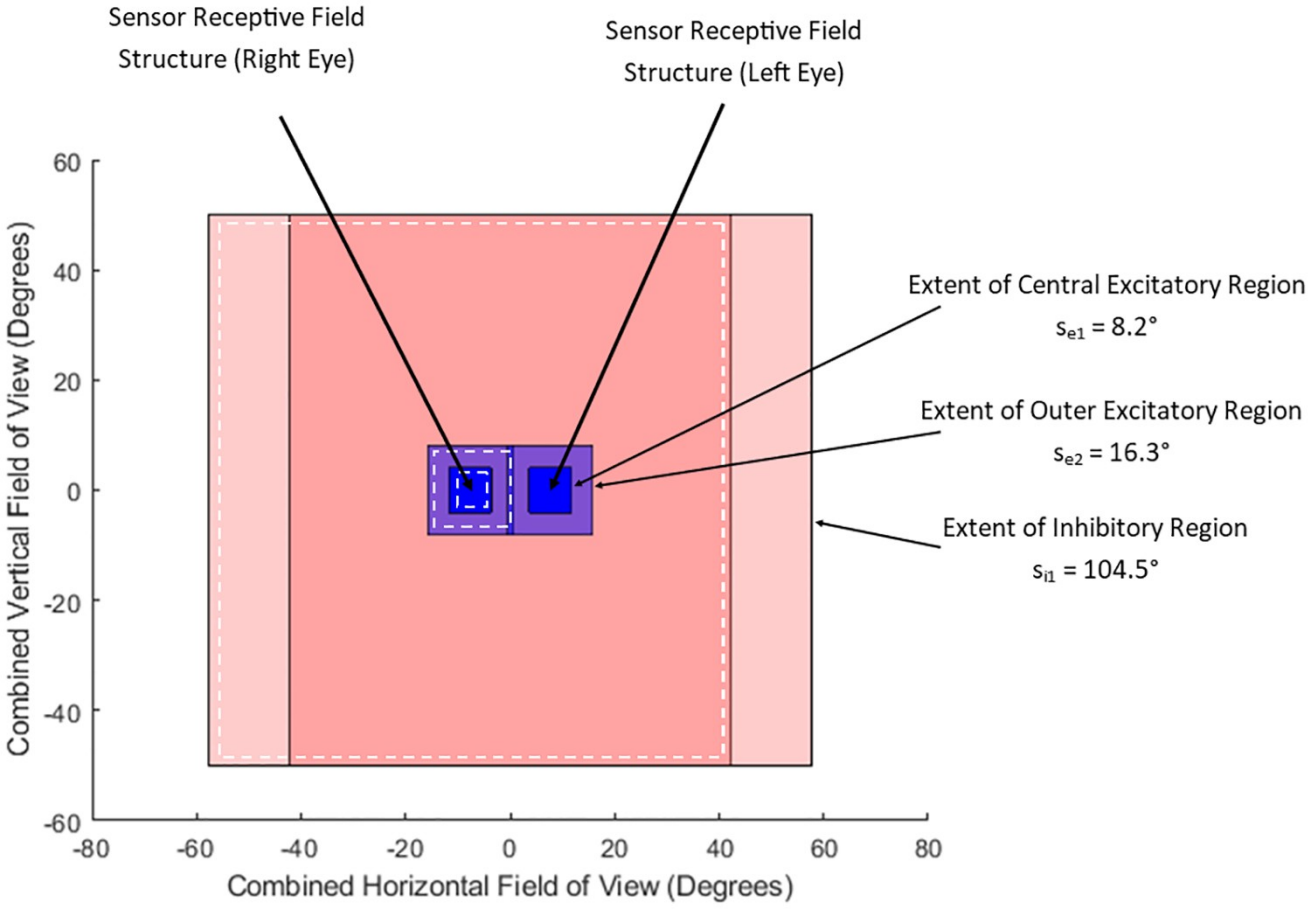
The image shows left- and right-eye receptive fields, superimposed and backprojected onto a screen at 10cm distance for ease of comparison with the stimuli. The left- and right-eye receptive fields have identical structures, with a central strongly excitatory region (blue) surrounded by an outer, more weakly excitatory region (purple), and then by a much larger inhibitory region (pink). The receptive fields in the two eyes appear offset horizontally on the screen by the screen disparity *d* = 15.4°.

The receptive field structure was assumed to be identical in the two eyes but offset horizontally; the amount of this offset controls the disparity tuning of the binocular neuron. We assumed that the receptive field in each eye had an excitatory centre and an inhibitory surround; the size of the excitatory region controls the preferred size of the neuron. For simplicity, our model receptive fields are constructed from square regions of uniform weight (Fig 6). We found that we needed to include both a central excitatory region with high positive weight and an outer excitatory region with lower positive weight, as well as an inhibitory surround with negative weight. Considering a monocular target centred on the receptive field (Fig 6), we can see that as the target size increases from zero, the total response will increase rapidly until the target fills the central, strongly excitatory region, and thereafter increase less rapidly until the target fills the outer excitatory region. As the target gets larger still, the response will decrease due to inhibition from the surround. When the target is large enough, inhibition from the surround will cancel out excitation from the centre, and there will be no output (Eq 2).

### 2.3 Parameter optimisation

**Fig 7:**
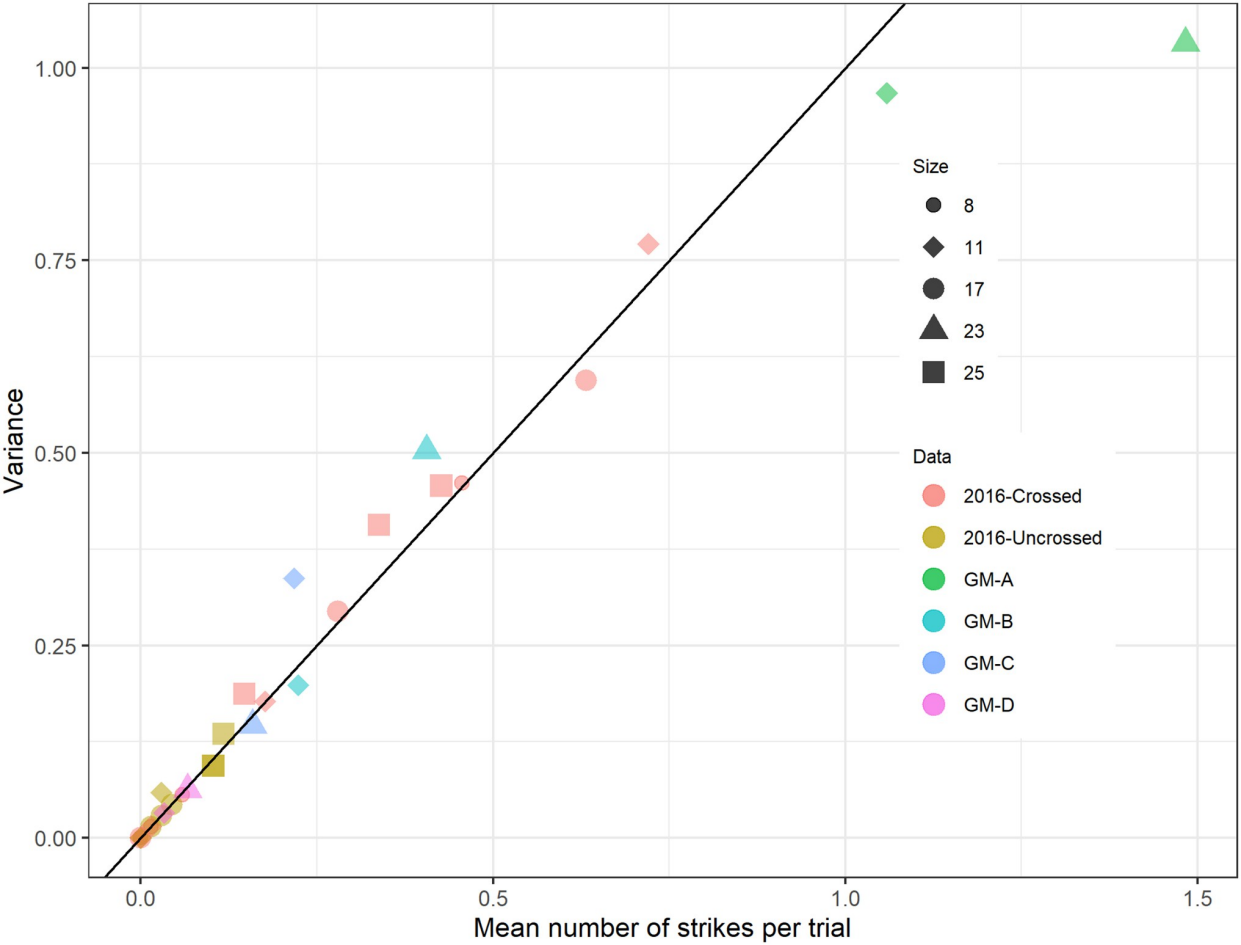
Variance versus mean for number of strikes per trial. Symbols labeled “2016-crossed/uncrossed” represent data from [10]; GM-A-D are from our ghost-match experiments, see Fig 10 below. We pooled data from all trials in all mantids for a given stimulus (disk size and geometry), and computed the mean and variance of the number of strikes observed across trials. The number of trials contributing to each data-point is 68 for the data from [10], 120 for the GM-D condition and 170 for GM-A,B,C. Symbols encode disk diameter in degrees; color encodes stimulus geometry and data-set. The scatterplot shows variance plotted against mean, with the black line marking the identity. For a Poisson distribution, variance = mean and so points should lie on the identity.

The model parameters are shown in Table 2. Those listed as “fixed” were set at the specified value, based on previous literature (or pilot experiments in the case of *s*_*i*_). Those listed as “optimised” were obtained by a maximum likelihood fit, comparing the expected number of strikes to the number empirically observed. We modelled the number of strikes as following a Poisson distribution. Fig 7 confirms that our empirical data is consistent with Poisson statistics, at least up to strike rates averaging 1 per trial, as in the dataset we are trying to fit. Thus, we sought the set of parameters **p** which maximised the function Eq 4:

$$\begin{array}{c} L\left(p\right)=\sum_{j}N_{trials}^{j}\left\{M_{data}^{j}\text{ln}\left[M_{model}^{j}\left(p\right)\right]-M_{model}^{j}\left(p\right)\right\} \end{array}$$ (4)

where the sum is over all stimulus conditions (varying in stereoscopic disparity and size); $N_{trials}^{j}$ is the number of trials conducted for that stimulus condition; $M_{data}^{j}$ is the mean number of strikes elicited per trial, averaged over all $N_{trials}^{j}$ trials; and $M_{model}^{j}\left(p\right)$ is the expected number of strikes per trial predicted by the model (Eq 3) for a stimulus at that simulated distance and size, given the current set of model parameters **p**. This expression is derived from the likelihood of observing *M*_*data*_ given a Poisson distribution with mean *M*_*model*_, though note that *L* is not exactly equal to the log-likelihood, since for simplicity we have omitted constant terms that do not depend on the model parameters and thus do not affect the optimisation. *N*_*trials*_ would be relevant if it varied between conditions, although in our data-set it was a constant. As expected, *L* is maximised as *M*_*model*_ approaches *M*_*data*_ for every trial.

**Table 2:** Parameters controlling the behaviour of our model, and how these were obtained. The top two rows are for the early visual system, and the remainder describe the disparity sensor. Parameters optimised by fitting to the data were constrained to lie between the stated bounds. The receptive field regions were squares, with the sizes *s*_*e*1_, *s*_*e*2_, *s*_*i*_ being the side-length. *s*_*i*_ is simply chosen to be very large compared to the stimulus. In our simulation, each pixel represents 0.154° visual angle. The sensor disparity is given both as a screen disparity *α*_*pref*_, and in parentheses as the corresponding distance *D*_*pref*_.

Symbol	Meaning	How determined	Lower bound	Upper bound	Value
N/A	standard deviation of Gaussian lowpass filtering	Fixed			0.6°
*τ*	time-constant of highpass filter	Fixed			20 ms
*α*_*pref*_	sensor position screen disparity, i.e. offset left- and right-eye receptive field centres, as measured on a screen at 10cm distance	Optimised	9° (3cm)	23° (1.5cm)	15.4°, (2.1cm)
*s*_*e*1_	size of receptive field central excitatory region	Optimised	3°	11°	8.14°
*s*_*e*2_	size of receptive field outer excitatory region	Optimised	11°	17°	16.3°
*s*_*i*_	size of receptive field inhibitory region	Fixed			104.5°
*w*_*e*1_	weight of receptive field central excitatory region	Optimised	0	+∞	6.77 × 10^−4^
*w*_*e*2_	weight of receptive field outer excitatory region	Optimised	0	+∞	3.18 × 10^−4^
*w*_*i*_	weight of receptive field inhibitory region	Optimised	−∞	0	−7.46 × 10^−5^
*b*	tonic inhibition onto disparity sensor	Optimised	−∞	0	−0.0542
*γ*	exponent of disparity sensor output power nonlinearity	Optimised	0	+∞	5.05

### 2.4 Stereoscopic distance and definitions of disparity

**Fig 8:**
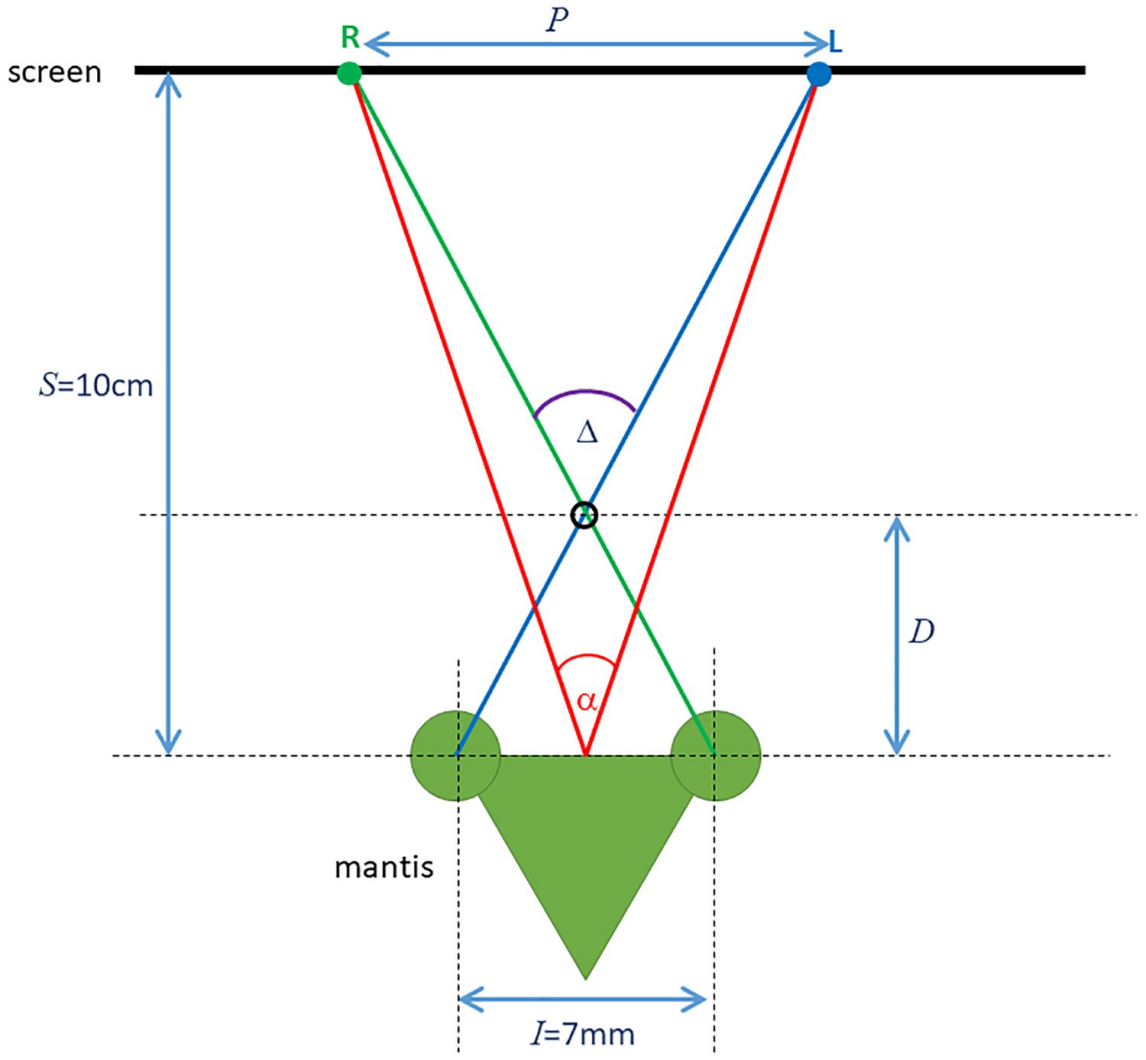
Top-down view (not to scale) of a mantis viewing a computer screen at a distance *S*. To simulate an object at distance *D* from the mantis, the left and right eye’s images (labelled L, R) need to be separated by a distance *P* on the screen. The angle subtended by the distance *P* at the mantis is the screen disparity *α*; the retinal disparity Δ of the virtual object is the angle subtended by the interocular distance *I* at the virtual object.

Our simulations are closely based on our behavioural and neurophysiological experiments, in which stimuli were presented on a screen 10cm from the animal’s eyes, as shown in Fig 8. Accordingly, we represent stimuli in the simulations as if they were being presented on a screen a distance *S* = 10cm away. Targets at a stereoscopic distance *D* are simulated by giving their images a horizontal separation *P* on the simulated screen, where

$$\begin{array}{c} P=\frac{I(S-D)}{D} \end{array}$$ (5)

Since we mainly measure image locations in degrees, we also express *P* via the angle *α* that it subtends at the eyes. We refer to this as *screen disparity*:

$$\begin{array}{c} \alpha =2\;\text{arctan}(0.5P/S) \end{array}$$ (6)

In our plots, we will display simulated images as they would have been presented on the screen. The separation between left- and right-eye images will thus correspond to the screen disparity *α*. A screen disparity of *α*, on a screen a distance *S* away from the animal simulates a virtual object at a distance *D*, where

$$\begin{array}{c} D=\frac{IS}{I+2S\;\text{tan}\;(\alpha /2)} \end{array}$$ (7)

and thus

$$\begin{array}{c} \text{tan}\left(\alpha /2\right)=\frac{I(S-D)}{2SD} \end{array}$$ (8)

*I* is the interocular separation, which we take to be 7mm throughout this paper. Biologically, the screen disparity *α* is not very relevant, since it reflects where we happened to position our screen in the experiments. A more relevant quality is the angle Δ, the *retinal disparity*. An object at distance *D* from the animal has retinal disparity

$$\begin{array}{c} \Delta =2\;\text{arctan}(0.5I/D) \end{array}$$ (9)

The relationship between screen disparity *α* and retinal disparity Δ is given by

$$\begin{array}{c} \text{tan}\left(\alpha /2\right)=\frac{\left(S-D\right)}{S}\text{tan}\left(\Delta /2\right) \end{array}$$ (10)

i.e. they become equal when the screen is at infinity. In this paper, we will mainly report the screen disparity *α* and the simulated distance *D*. To recover retinal disparity Δ, use the above expressions with our simulated interocular distance *I* = 7mm and screen distance *S* = 10cm.

### 2.5 Stimuli

#### 2.5.1 Simulated behavioural experiments

For optimising model parameters and exploring the behavioural response, we use stimuli consisting of bright disks, pixel value 1, on a dark background, pixel value 0 (in fact mantids prefer dark stimuli on a bright background, but the model presented here is only sensitive to changes in contrast, not the direction of the change, and so this does not affect our results). In most of our behavioural experiments, including the source of the data used for fitting [10], we used stimuli which moved in a spiral trajectory from the outer edges of a monitor screen to the centre. The target began more than 100° away from the centre of the screen, moving with a velocity of almost 1500° per second, and gradually decelerates over 5 seconds, ending with a velocity of 0 in the centre of the screen.

This stimulus is ill-suited for understanding the properties of our disparity-tuned model neuron, since the response will be affected by the speed and direction of the stimulus, both of which are constantly changing in the spiralling stimulus. In this paper, we therefore chose to examine targets moving with a constant speed either horizontally or vertically. These two cardinal directions are the most important to examine, since the binocular receptive fields are offset horizontally but aligned vertically, and so the response is expected to be different for these cases. For our simulations, we chose a constant stimulus speed of 82° per second. Previous work has shown that this speed is close to optimal for eliciting mantis strikes [32], and we have successfully elicited strikes with stimuli moving horizontally at this speed [21]. The spiralling stimulus of [10] reached this speed when it was 15° from the centre of the monitor.

### 2.6 Empirical data

We optimised model parameters using the data from our earlier paper, [10], which is available at http://dx.doi.org/10.1098/rstb.2015.0262. The data for stimuli simulating distances within catching range were shown in Fig 4. In the paper, as a control, we also presented stimuli with equivalent uncrossed disparity, as in Fig 1B. Uncrossed stimuli were identical to stimuli used to depict a given virtual distance, except that left and right images were swapped. In uncrossed stimuli, the strike rate was unsurprisingly very low. However, a strike rate of around 10% was observed for the largest stimuli (25° diameter). Because striking was independent of binocular disparity in uncrossed stimuli, we concluded that this was most likely a defensive behaviour, aimed not at catching prey but at deterring a potential threat, and triggered by the angular size of the stimulus rather than its disparity. We would therefore not expect such strikes to be under the neural control of a sensor designed to detect prey within catch range, which is what we are trying to simulate here.

**Fig 9:**
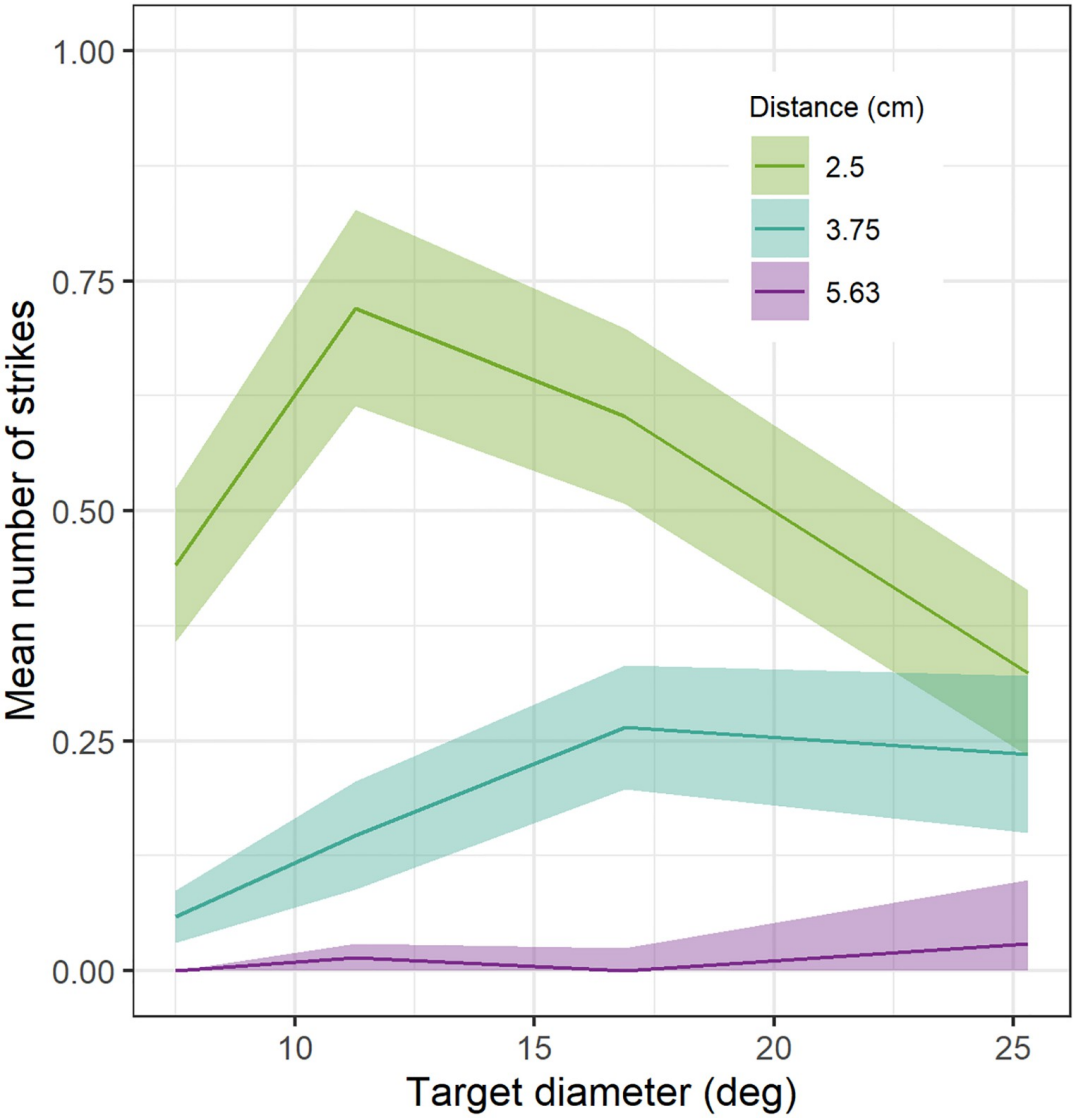
Modified behavioural response of mantids. Mean number of strikes [10] in response to different simulated distances, as a function of the angular size of the simulated target in the crossed condition, with strikes in the corresponding uncrossed condition subtracted. Ribbons show ±1 standard error on the mean.

Accordingly, we corrected the raw number of strikes by subtracting strikes in the uncrossed condition from strikes in the equivalent crossed condition. The results are shown in Fig 9.

For optimising model parameters, we also included synthetic data for monocular, distant and large targets which were not included in our experiments precisely because in our experience they elicit very few strikes (Table 3). These conditions were assigned a strike rate of 0, forcing the optimisation to avoid parameter sets which would predict implausibly large values there. *N*_*trials*_ was set equal to 68 for these conditions too, giving them the same weight as the other conditions.

**Table 3:** Values of *M*_*data*_ (mean number of strikes per trial) used for optimisation. Columns show stimulus diameter; rows show stimulus stereoscopic distance. For the top 3x4 cells (distances 2.5-5.63cm and sizes less than 38°), data was derived from [10] by taking the difference between the total number of strikes recorded for crossed vs uncrossed trials for the given stimulus, divided by the total number of times each stimulus was presented (*N*_*trials*_ = 68). This was non-negative in all cases except distance 5.63cm and size 16.88°, where it was -0.04 Zeros for size = 38°, distance = 10cm and monocular stimuli are synthetic data included to force the optimisation to choose parameter values which predict very low strike rates for these conditions, in keeping with our observations. [10].

	7.5°	11.25°	16.88°	25.31°	38.00°
2.50*cm*	0.44	0.72	0.60	0.32	0.00
3.75*cm*	0.06	0.15	0.26	0.24	0.00
5.63*cm*	0.00	0.02	0.00	0.03	0.00
10*cm*	0.00	0.00	0.00	0.00	0.00
Monocular	0.00	0.00	0.00	0.00	0.00

As noted above, in our mantis experiments stimuli moved in a spiral trajectory with varying speed and direction, and strikes could be triggered potentially at any point. In our simulations, stimuli moved in a straight line with constant speed. For optimising our model parameters, we used stimuli which passed through the centre of the receptive field (i.e. the average of the left and right receptive fields, given that these were offset horizontally), with zero vertical disparity and a horizontal disparity corresponding to the simulated distance condition. We ran each condition twice: once for vertical motion and once for horizontal. The model’s prediction for the mean number of strikes, *M*_*model*_, depended on the direction of motion, and we included both conditions in the sum describing our cost function Eq 4, using the same *M*_*data*_ for both as specified in Table 3.

### 2.7 Simulation details

**Fig 10:**
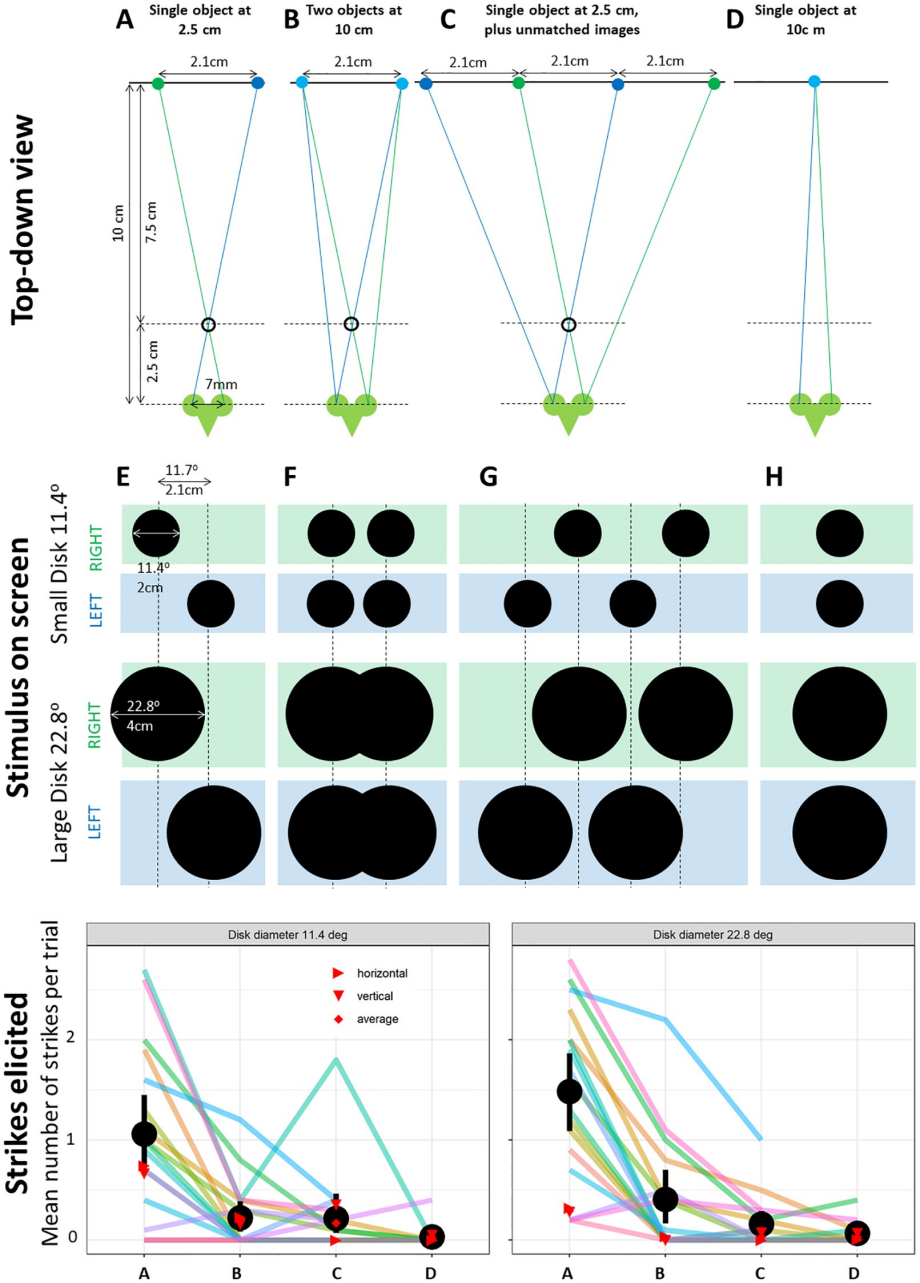
Testing the response of model and mantids to ghost matches. Top row shows four different stimulus geometries: (A) a single object 2.5cm distant from the mantis, simulated by disparate objects on a screen 10cm distant, with screen parallax 2.1cm; (B) two objects 10cm distant, 2.1cm apart; (C) as A but with an additional image presented to each eye, with diverging lines of sight meaning that these cannot correspond to any one physical object; (D) single object 10cm distant. Middle row indicates how each of these appear on the screen as viewed by the right and left eye, for the two different disk diameters used. Bottom row: Mean number of strikes elicited per trial for each size and stimulus geometry. Coloured lines: average over 10 trials for each of 18 mantids (not all mantids were tested on the control condition D). Black dots: group average over all mantids; errorbars, ± standard error. Red symbols: mean number of strikes predicted by the model, for horizontal motion (⊳), vertical motion (▽), and for the average of the two (◊).

All simulations were carried out in Matlab R2019a (www.themathworks.com). For examining the behavioural responses, we generated the stimuli in each eye as images of 680 × 680 pixels, with each pixel representing approximately 0.15° visual angle. Each stimulus consisted of, in each eye, a light disk (pixel value 1) on a dark background (pixel value 0) (or a pair of two such disks for the ghost-match stimuli, see Fig 10B and 10D). The simulation timestep was fixed at 3.3 ms (300Hz), chosen to be substantially lower than the 20ms time-constant of the temporal filter to avoid numerical artefacts. Since the images in our experiments [10] were presented on an LCD monitor with a refresh rate of 60Hz, we updated the images in our simulation every 16.66 ms, i.e. each frame was presented to the model 5 times before the target advanced in the next frame. In each frame update, the target advanced 9 pixels = 1.37° either horizontally or vertically, giving the desired speed of 82°/*s*. The relative position of the left- and right-eye disks remained fixed.

The horizontal position of the target in each eye was constant for targets moving vertically, and the vertical position was constant for targets moving horizontally. We investigated various choices for these, as discussed in the text. The size of the disk also varied, as specified in the relevant figures. In each case, stimuli crossed the entire visual field of our model, spanning a visual angle of 60°.

The input images were then spatially filtered using the function ‘imgaussfilt’ from the Matlab image processing toolbox, with a Gaussian filter with parameter SD set to 4 (pixels). The SD represents the acceptance angle of the ommatidia, which was approximated at 0.7° [40]. These filtered images were then passed through a first-order high-pass temporal filter using Matlab function ‘filter’. Finally, the output of the temporal filter was squared.

These filtered outputs then formed the inputs to the receptive fields of the disparity-tuned neuron. Eq 1 was implemented by point-multiplying each output frame with the model receptive field function in each eye, and summing the result across both dimensions.

After combining the results from both eyes according to Eq 2 at each sampled time-point, Eq 3 was implemented using the Matlab function ‘trapz’. This produced a value of *M*_*model*_ to use in the optimisation cost function, Eq 4. We did this twice for each condition, once for a vertically-moving target and once for horizontally, and added both onto the cost function.

### 2.8 Parameter optimisation

The model parameters in Table 2 were optimised via gradient descent using the Matlab function ‘fmincon’, with −*L* (Eq 4) as the function to be minimised. fmincon finds the minimum of a constrained nonlinear multivariable function; we constrained all model parameters to have a lower bound of 0 with the exception of the bias parameter *b* (Eq 2), which was constrained to be negative, between -∞ and 0.

The choice of starting point is often critical in multi-dimensional optimisation problems such as this. We used initial values chosen at random within the upper and lower bounds for each parameter (see Table 2). The upper and lower bounds were selected non-randomly, to reflect our own prior knowledge of the sensor where possible. For example, since we know that the strike rate is highest for distances around 2.5cm, we constrained the sensor disparity *d* to correspond to distance in the range 1.5cm to 3cm. In the process of obtaining the parameter values we settled upon and present in this work, the optimisation process was performed many times (before and after the presented parameter set was found) and each parameter with non-infinite upper and lower bounds was tried with starting values spanning the range of the possible values it could assume, to improve the chances of finding the true global optimum.

### 2.9 Behavioral experiments

Most of the behavioral data used in this paper has been published previously, and readers are referred to the cited publication for methods. Table 4 and Fig 10 show new data from a different set of mantids. In these experiments, the relative positions of all disks remained constant while the pattern as a whole moved in the spiralling motion described previously [10]. We ran all four conditions both with small disks, of 11.4° diameter, and large ones, 28.4°. Each of the 8 stimuli were presented 10 times to each of 18 mantids, *Sphodromantis lineola*, with the different conditions randomly interleaved.

**Table 4:** Mantids are not fooled by ghost matches. Mean number of strikes predicted by the model, and observed in mantids, for the four stimulus geometries shown in Fig 10A–10D.

Disk size	Results from:	Motion	A	B	C	D
11.4°	Model	Horizontal	0.67	0.16	0.36	0.05
	Model	Vertical	0.74	0.23	0.00	0.00
	Model	Average	0.70	0.20	0.18	0.03
	Mantis group mean	Spiral	1.06	0.22	0.22	0.03
22.8°	Model	Horizontal	0.28	0.00	0.08	0.07
	Model	Vertical	0.32	0.04	0.00	0.01
	Model	Average	0.30	0.02	0.04	0.04
	Mantis group mean	Spiral	1.48	0.41	0.16	0.07

We also ran model simulations for the stimulus conditions shown in Fig 10. The target motion was either vertical or horizontal, as described above. For vertical motion, the horizontal position of each disk was fixed as in Fig 10A–10D, so that the stimulus as a whole was symmetric about the midline *x*_*c*_ = 0. For horizontal motion, the vertical position of each disk was fixed at *y*_*c*_ = 0. Again, the relative positions between disks remained constant.

## 3 Results

### 3.1 Predicted striking behaviour as a function of target size and binocular disparity

**Fig 11:**
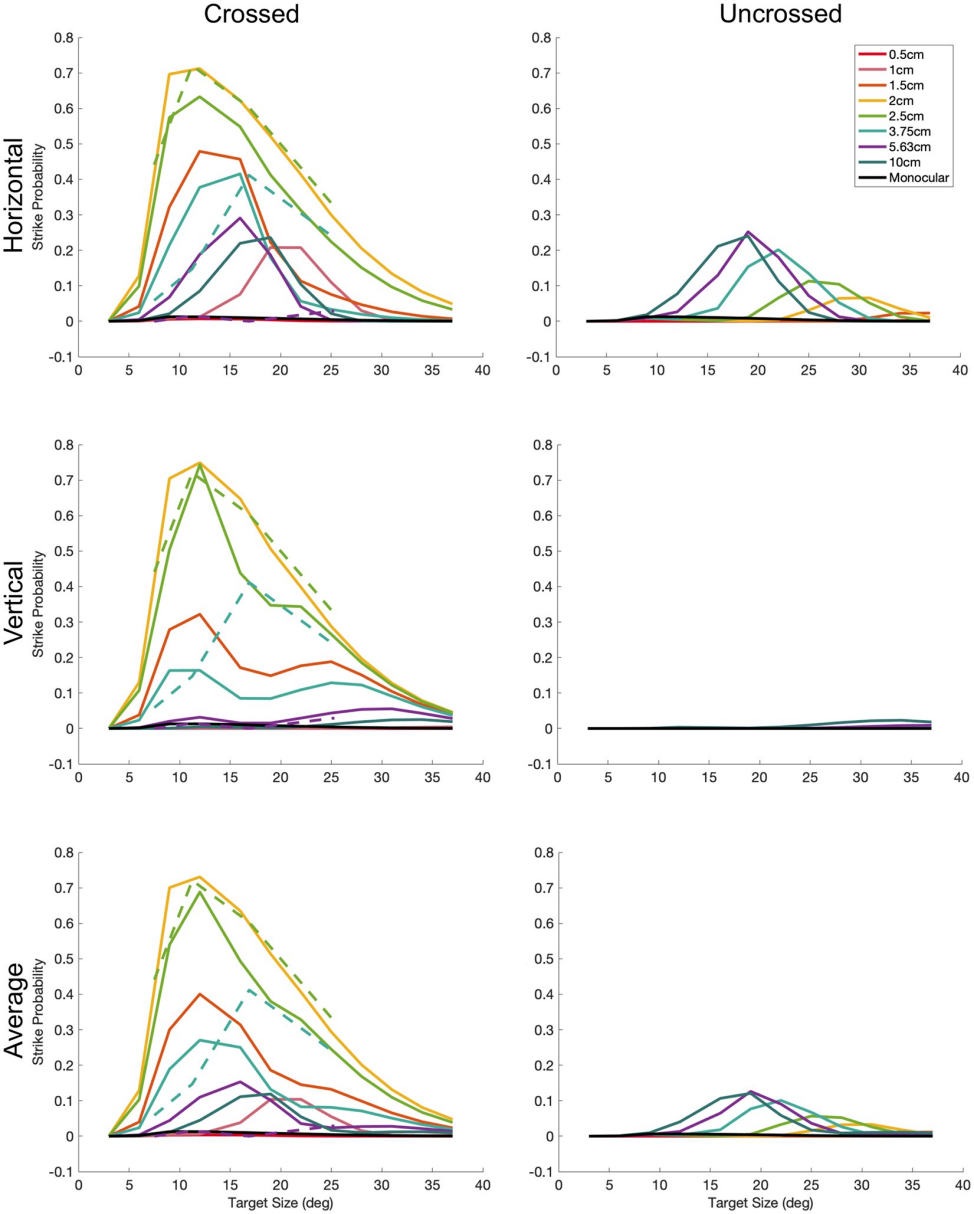
Mean number of strikes per trial predicted by the fitted model, as a function of stimulus size (horizontal axes) and simulated distance (colours). The top row is for targets moving horizontally over the sensor; the middle for stimuli moving vertically, and the bottom row for the average of both. The left panels are for crossed stimuli and the right for uncrossed. In all cases, the target passed directly over the receptive field center. The dashed lines in the crossed panels show the empirical data from [10] (Table 3) which the optimisation aimed to reproduce. In the experiments, the target had a spiralling motion with both horizontal and vertical components, so the same empirical data are shown in all three rows. As described in the Methods (see Table 3), the optimisation also aimed to predict zero strikes for stimuli at 10cm simulated distance, for monocular stimuli, and for stimuli of size 38° diameter. The other conditions—intermediate sizes and disparities, and uncrossed stimuli—were not constrained in the fitting, but the model structure ensures that plausible results are also obtained for these.

Fig 11 shows the predictions of the fitted model (Table 2) for the expected number of strikes per trial, as a function of target size and distance, compared with empirical results (dashed lines). The results are broadly independent of target direction of motion and broadly in line with empirical results despite the simplicity of the model. Although the peak response in the empirical data was at 2.5cm, the optimisation finds the best match with receptive fields whose binocular position disparity corresponds to a distance of 2.1 cm. Accordingly, the curve for 2cm has the highest peak, with curves for nearer and further distances eliciting a lower response, in agreement with mantis behaviour. The asymmetry in the effect of disparity on strike probability reflects the geometry: moving from a distance of 2cm to 2.5cm involves moving the target laterally 1.5° in each eye, whereas moving from 2cm to 1.5cm involves a larger shift of 2.6°. This produces a lower response, as more of the target moves into the inhibitory surround of the receptive field. The model also captures the asymmetry in size tuning, with response falling more slowly as target diameter increases beyond the preferred size than when it decreases below it.

For targets at the preferred simulated distance of 2.1cm, the predicted strike rate is the same regardless of target direction of motion. However, for other distances, direction of motion makes a difference. This anisotropy reflects the fact that the receptive fields have horizontal, but no vertical, disparity.

Most notably, the model predicts some strikes to large uncrossed stimuli moving horizontally, but virtually none to vertically (cf Fig 11, middle row). No one has yet compared the effect of motion direction on mantis strikes uncrossed stimuli, so we do not know if any such anisotropy occurs empirically. We have previously observed a strike probability of 10-12% for large uncrossed targets ([10], their Fig 5a), but this effect did not depend on target disparity, and so, as noted above, we concluded it was not driven by the stereoscopic system.

For horizontal motion, the model also shows an interaction between size and disparity tuning. The angular size eliciting the most strikes is 20° for targets 1cm away, then decreases to 12° for targets 1.5cm, then decreases further to 10° for targets 2-2.5cm away, then increases again for more distant targets. The data of [10] showed a qualitatively similar effect.

As we discuss in the next section, the underlying reason for both of these effects is that, for stimuli moving horizontally, it is possible for the leading edge of the disk in one eye to stimulate the sensor at the same time as the trailing edge in the other eye.

### 3.2 Explanation of model behaviour

**Fig 12:**
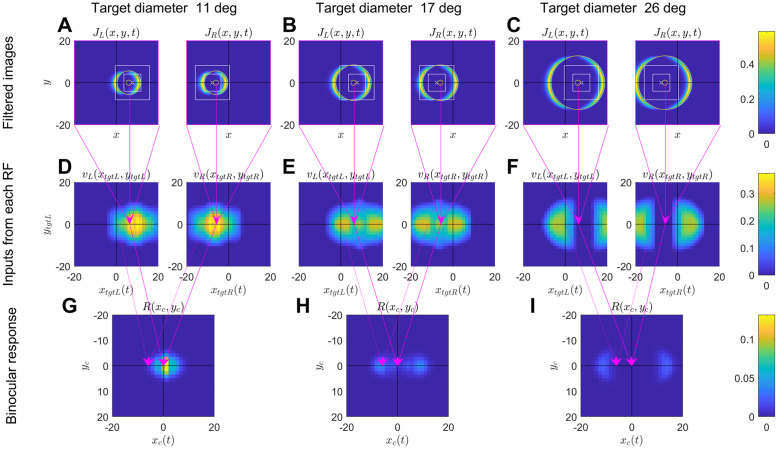
Responses of different model components to a horizontally-moving disk at a simulated distance of 2.5cm from the mantis. Left, middle, right columns are for a target of size 11.2°, 16.9° and 25.5° as indicated. Sub-panels in the top two rows show left and right eyes. In all panels, axis coordinates are in degrees visual angle referred to the centre of the screen, i.e. *x* = 0, *y* = 0 corresponds to a location 10cm directly in front of the mantis. However, the interpretation of the axes differs in each row, as explained below. Top row (ABC): Snapshots of the filtered images *J*_*L*,*R*_(*x*, *y*, *t*), shown as a function of retinal location (*x*, *y*) for one particular time *t*. The axes are therefore simply retinal location. Pseudocolor represents the images reaching the sensor’s receptive fields, following lowpass spatial filtering, highpass temporal filtering and squaring in the early visual system. The receptive field excitatory region is shown superimposed for comparison. Each pixel represents the value of the filtered image at a particular location in the retina. These snapshots are for one particular time *t* and thus for one particular target position *x*_*tgt*_(*t*), *y*_*tgt*_(*t*) as the target moves across the screen. In this figure, the target was moving horizontally, so *y*_*tgt*_ is in fact independent of time whereas *x*_*tgt*_ = *x*_0_ + *Vt*. The yellow circle marks where the center of the target is in that eye at the time shown. The white cross marks the center of the sensor receptive field in that eye; the inner white square marks the boundary of the central excitatory region, while the outer white square marks the boundary of the outer excitatory region. The surrounding inhibitory region extends beyond the range shown in each panel. Thus, parts of the filtered image falling outside the white squares have an inhibitory effect on the sensor. Middle row (DEF): Inputs to the binocular disparity sensor from the two eyes, *v*_*L*,*R*_. The input from each eye is the inner product of the monocular receptive field with the filtered image at that moment in time. It is here represented as a function of target position *x*_*tgt*_(*t*), *y*_*tgt*_. Since the target is moving horizontally across the screen from left to right, *x*_*tgt*_ is a function of time, whereas *y*_*tgt*_ is constant for a given trajectory. Each pixel-row in DEF therefore represents the time-course of the monocular input, *v*_*L*,*R*_(*t*), as the target moves from left to right over the screen, at the vertical location *y*_*tgt*_ corresponding to the height of the pixel row. The axes therefore represent the current location of the target in the retina, and the panel as a whole does not represent an image, since different locations correspond to different times. The pink arrows mark the value of the monocular input in D for the filtered image shown in A. Bottom row (GHI): response of the disparity sensor, Eq 2. The axes are now the current visual direction of the moving binocular target, *x*_*c*_(*t*) = 0.5(*x*_*tgtL*_(*t*) + *x*_*tgtR*_(*t*)); *x*_*c*_ is again a function of time. Arrows from D to G show the target locations shown in the top row, A, and thus the response when the target crosses the midline, *x*_*c*_ = 0. For comparison, dotted arrows show the response a little earlier when *x*_*c*_ was −6°. The target’s direction in the visual field is *x*_*c*_ = (*x*_*tgtL*_ + *x*_*tgtR*_)/2 and *y*_*c*_ = (*y*_*tgtL*_ + *y*_*tgtR*_)/2. Since the target is moving horizontally, *x*_*c*_ is a function of time, but *y*_*c*_ is constant for a given trajectory.

In any computer model, it is important to understand how the model achieves its results: what features are key to the behaviour (e.g. centre/surround receptive field structure) and which are secondary (e.g. receptive fields modelled as squares). In this section, we dive under the hood of the model in order to understand in detail how it produces its results. Fig 12 shows the response of different components of the model when the virtual stimulus is a disk of size 11° (A), 17° (B) and 26° (C), moving horizontally from left to right across the screen at a simulated distance of 2.5cm. The top row shows the filtered images in left and right eyes, the middle row the inputs to the disparity sensor from left and right receptive fields, while the bottom row shows the response of the disparity sensor.

#### 3.2.1 The filtered images

Before understanding the response of the disparity sensor itself, we first need to understand what is happening in each eye. The top row of Fig 12 shows snapshots of the filtered image, *J*(*x*, *y*, *t*), at the instant where the disk crosses the midline. Superimposed on this for reference, a large yellow circle marks the edge of the target at the instant shown; the small yellow o marks its centre. The target disk is simulated at a distance 2.5cm in front of the mantis, corresponding to a screen disparity of 12°. At the moment shown, the disk is centered on *x*_*tgtL*_ = 6° in the left eye and *x*_*tgtR*_ = −6° in the right, so that its overall direction is straight ahead (*x*_*c*_ = (*x*_*tgtL*_ + *x*_*tgtR*_)/2 = 0).

Because of the high-pass temporal filtering, the filtered image appears as two crescents, lagging behind the leading and trailing edges of the disk as it passes over the photoreceptors from left to right. In general, the time that elapses between the leading and trailing edges is *T* = *s*/*V*, where *s* is the target diameter and *V* its speed. If *T* is large compared to the timeconstant *τ* of the highpass filter, leading and trailing edges will be clearly separated and will have the same amplitude. The width of each edge will be proportional to speed *V*. As *T* decreases, the amplitude of the trailing edge relative to the leading one will decrease as [1 − exp(−*T*/*τ*)]^2^. Eventually, for *s* < *Vτ* ln(2) the edges will merge, without any dip between the leading and trailing edges. In this paper, we use a timeconstant of *τ* = 20ms and a target speed of *V* = 82° per second. The edges are therefore smeared over roughly 1.64°, and they do not merge except for diameters below 1°.

The central and outer excitatory regions of the disparity sensor’s receptive fields are also superimposed for comparison in Fig 12A–12C (concentric white squares). The center of the receptive field is marked with a white cross; it is at *x*_*L*_ = +7.7° in the left eye and *x*_*R*_ = −7.7° in the right, giving it the sensor screen disparity of *α*_*pref*_ = 15.4°. The disparity sensor is thus tuned to a location 2.1cm directly in front of the mantis. Recall that surrounding the excitatory regions shown is a much larger inhibitory region. The 3.4° relative disparity between the sensor (screen disparity *α*_*pref*_ = 15.4°) and the target (screen disparity 12°) explains why the target is at different locations relative to the receptive fields in the two eyes.

#### 3.2.2 Inputs from each eye’s receptive field

As we saw in Eq 1, the instantaneous input from each eye to the binocular sensor, *v*_*R*_(*t*) and *v*_*L*_(*t*), is the result of point-multiplying the receptive field function with the filtered image *J*(*x*, *y*, *t*) at that instant. Thus, for each subpanel in Fig 12A–12C, there will be a single value of *v*_*L*,*R*_(*t*) at the time of that snapshot. Its value will of course depend on where the target is located relative to the receptive fields at the moment of the snapshot.

This is illustrated in Fig 12D–12F. Here, each subpanel shows the values of *v*_*L*,*R*_ as a function of the current target position in that eye. The pink arrows linking each subpanel of Fig 12A–12C to a pixel in the corresponding subpanel of Fig 12D–12F show where the example images shown in Fig 12A–12C are represented in Fig 12D–12F. Other pixels in the same row of Fig 12D–12F would represent the value of *v*_*L*,*R*_ at different points in the target’s trajectory. Reading across a single pixel row in Fig 12D–12F, we can see how the values of *v*_*L*,*R*_ change as the disk crosses the receptive field. This is why the x-axis is labelled *x*_*tgt*_(*t*), since the x-position of the target is changing with time. A horizontal trajectory with a different y-location, higher or lower on the screen, would be represented by a different pixel-row Fig 12D–12F, with the appropriate value of *y*_*tgt*_.

For the smallest disk shown, Fig 12D, there is a single central peak. This occurs when the target is moving across the excitatory regions of the receptive field. At the moment shown in Fig 12A, in the left eye, activation caused by the leading edge of the disk is in the central excitatory region, where excitation is strongest. The trailing edge is mainly in the outer excitatory region, but some is still in the inhibitory region. In the right eye, both leading and trailing edges are mainly in the outer excitatory region, causing weak excitation but no inhibition. As we see from the corresponding pixel values in Fig 12D, the net effect is similar in both eyes. Because the 11° disk is small enough that both leading and trailing edges fit comfortably within the excitatory region, there is a single peak as the disk passes across the receptive field.

Conversely for the 17° disk, activation is a little weaker at the moment indicated with the arrows in Fig 12E. As we see from Fig 12B, in the left eye the leading edge has just passed out of the central excitatory region into the outer excitatory region where excitation is weaker, while the trailing edge is still in the inhibitory surround. So, the corresponding value of *v*_*L*_, pointed to by the arrow in Fig 12E, is lower than it was a moment ago. It will shortly rise again when the trailing edge enters the excitatory region. For the 17° disk, there are thus two distinct peaks of activity in *v*_*L*,*R*_, which occur when first the leading and then the trailing edge of the disk cross the central excitatory region.

For the largest disk shown, in Fig 12C, 12F and 12I, there are also two peaks, but this time the central dip between them is much lower—in fact slightly negative—because there is a time when both edges are almost entirely within the inhibitory surround (cf right eye in Fig 12C). The peaks on either side of the dip are also lower. This is because the larger disks don’t fit entirely within the excitatory region. When the leading edge is crossing the central excitatory region, the trailing edge is still in the inhibitory surround. Because the weight of the central region is stronger, there is still net excitation, but less than for the smaller disk. In fact, this disk is too large for even one edge to fit within the excitatory region: the top and bottom are always within the inhibitory surround. As expected given its centre/surround structure, the monocular receptive fields are size-tuned, responding maximally to disks around 11° in diameter. This is why the peak value of *v*_*L*,*R*_ is larger in Fig 12D than in Fig 12F.

#### 3.2.3 Binocular response

We now consider the binocular response. The bottom row of panels shows the output of the disparity sensor, *R* = ⌊*v*_*L*_(*t*) + *v*_*R*_(*t*) + *b*⌋^*γ*^ (Eq 2), with the fitted values *b* = −0.054 and *γ* = 5.05. Instead of plotting this directly as a function of time, we have shown it as a function of *x*_*c*_(*t*) = 0.5(*x*_*tgtL*_(*t*) + *x*_*tgtR*_(*t*)), the visual direction of the target. Again, because the target is moving from left to right at constant speed, these two representations are equivalent. Values of *x*_*tgtL*_(*t*) and *x*_*tgt*,_(*t*) for given *t* always differ by the screen disparity of the target, here 12°. The arrows from Fig 12D to 12G show two examples. The solid arrows show the value of *R* when the target crosses the midline, *x*_*c*_ = 0°, while the dotted arrows are for the earlier time when *x*_*c*_ = −6°.

**Fig 13:**
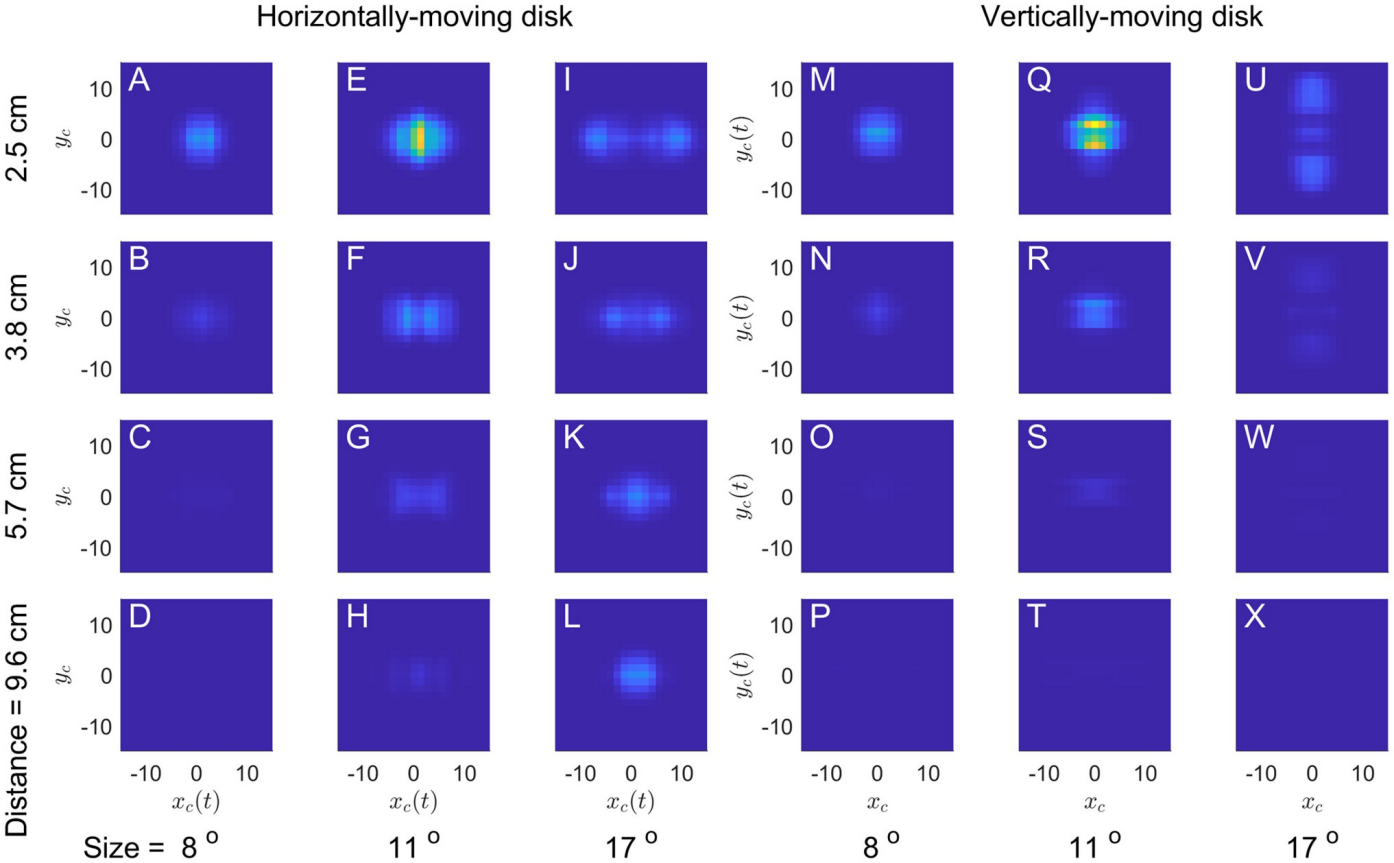
Model sensor output *R*(*x*_*c*_, *y*_*c*_) for horizontally or vertically travelling targets as they move across the centre of the field of view. Different rows are for different target distances, as indicated down the left-hand side of the figure, while different target sizes are in columns, as indicated along the bottom. The pseudocolour shows the instantaneous response of the disparity sensor as the centre of the moving target first reaches the location indicated by the (x,y) position. (To simulate the 60Hz refresh rate of the monitor, the target changes position every 16ms, and so stays in each location for five of the simulation’s 3.3ms timesteps). The color axis is the same in all 24 panels.

The expected number of strikes for a given trajectory is found by integrating the response along the corresponding row of the panel. Thus, the strike rate depends not only on the peak value, but also on the number of peaks. For the parameter optimisation and for the results shown in Fig 11, the target passed directly over the receptive field center, corresponding to the trajectory with *y*_*c*_ = 0. (As Fig 13 makes clear, if the target does not pass directly over the center, the response is reduced but the dependence on size and disparity remains similar, so the restriction to targets passing directly over the receptive field does not lose behaviour of interest).

The high value of the output exponent *γ* makes the sensor extremely sensitive to the value of the combined receptive field outputs, *v*_*L*_ + *v*_*R*_. This enhances the size tuning and also ensures that the sensor is both very sensitive to disparity and unresponsive to monocular stimuli. At the time of the snapshot in Fig 12A, the inputs to the sensor from each eye, *v*_*L*_ and *v*_*R*_, are each close to their peak value, roughly 0.35. The sensor response is therefore (0.35 + 0.35 − 0.054)^5.05^ = 0.11.

However, if the inputs to the sensor halve, the sensor output falls by a factor of 50 ((0.35 − 0.054)^5.05^ = 0.002). The fairly small reductions in receptive field output as the disk size increases are thus greatly exaggerated in the disparity sensor (compare Fig 12D–12F with Fig 12G–12I). The same effect means that there is virtually no response to monocular stimuli, where the peak input is necessarily halved.

#### 3.2.4 Disparity tuning

**Fig 14:**
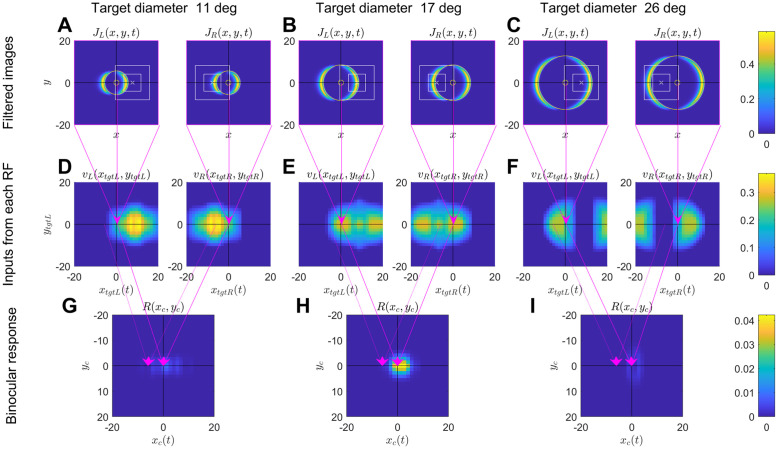
As for Fig 12, but for a target at a simulated distance of 10cm from the mantis (zero screen disparity).

Fig 14 shows the effect of altering the binocular disparity. It is the same as Fig 12, except for a disk on the screen plane, at a distance of 10cm, instead of at a simulated distance of 2.1cm (and with a different color axis for the binocular response row). The target screen disparity is now zero: that is, the left and right half-images always appear at the same locations on the screen, making them offset from the sensor receptive fields. As is clear from comparing Fig 14 with Fig 12, the monocular time courses are unchanged, but they are shifted relative to one another. The peak response to the 11° disk is thus greatly reduced: when the right-eye input is maximal, the left-eye input is minimal. The maximum input to the binocular sensor is therefore reduced, and the output exponent *γ* amplifies this further, so the sensor response is very weak (dotted arrows linking Fig 14D and 14E to 14G). The strongest response is elicited when both inputs are medium (solid arrows) but this response is far weaker than when the target disparity matched the sensor disparity (compare Fig 14G with Fig 12G). Since strike probability reflects the activity of this sensor integrated over time, this weaker response explains the much lower strike rates shown in Fig 11 (70% strike probability for an 11°-disk at 2cm, compared with <10% at 10cm).

#### 3.2.5 Disparity-dependent size tuning

As discussed above, Fig 11 also shows the interesting property that the preferred target size varies with disparity. Targets 2cm from the mantis produce the most strikes if they are around 10° in diameter. However, targets 10cm from the mantis produce the most strikes if they are around 17° in diameter. Fig 14 explains why this occurs.

For the 17° disk, the separation of leading and trailing edges is close to the difference between the disparity of the sensor receptive fields and that of the target (*α*_*pref*_ = 15.4°). This means that the leading edge in the left eye crosses the central excitatory region of the left-eye receptive field at the same time as the trailing edge in the right eye crosses the central excitatory region of the right-eye receptive field (Fig 14B). Accordingly, the binocular sensor receives strong input from both eyes simultaneously (solid arrows from Fig 14E to 14H). This explains why there is a relatively strong response in Fig 14H at *x*_*c*_ = 0. It is due to a “false match” between the leading edge in the left eye and the trailing edge in the right eye. This response is still weak compared to the response to the optimal size and disparity (note the different colorscale in Fig 14G–14I vs Fig 12G–12I) but it does explain the shift in size tuning with disparity that we see both in the empirical data and in the model (Fig 11). As the target passes in front of the mantis, there is only one false match (leading-trailing), whereas when the target is at 2.1cm there are two true matches (leading-leading and trailing-trailing; cf double peaks in Fig 12H). Thus the true matches always produce the highest activity averaged over time, and so at all sizes, the highest strike rate is obtained when the target is at 2.1cm (the disparity tuning is not size-dependent, Fig 11). But the false match possible for large targets explains why the size-tuning is disparity-dependent.

#### 3.2.6 Effect of direction of motion

False matches between leading and trailing edges also account for the weak but non-zero strike rates predicted for uncrossed stimuli. Because the receptive fields have only horizontal disparity, not vertical disparity, false matches between leading and trailing edges can occur only for horizontal target motion. This explains why the model predicts disparity-dependent size tuning for crossed targets, and strikes to uncrossed stimuli, only for targets moving horizontally (Fig 11A vs 11B).

Fig 13 shows the sensor response for different sizes, disparities and directions of motion, in the same way as above for Fig 12G–12I. The color of each pixel represents the instantaneous response as the disk passes the location (*x*_*c*_, *y*_*c*_). For the disks moving horizontally (Fig 13A–13L), *x*_*c*_(*t*) increases linearly with time whereas *y*_*c*_ is constant; thus each pixel row in a subpanel shows the time-course of the response for a disk at the given *y*-position. For the disks moving vertically (Fig 13M–13X), *y*_*c*_(*t*) increases linearly with time whereas *x*_*c*_ is constant, so here each pixel column shows the time-course for a disk at the given *x*-location. Fig 13E and 13I correspond to the horizontally-moving disks at 2.5cm which were shown in Fig 12G and 12H, with diameters 11 and 17° respectively.

For a disk at 2.1cm, corresponding to the sensor disparity *d* (Table 2), the response would be identical for both directions of motion. This is because in this case, the target passes exactly through the middle of the receptive field in each eye, at the same time in both eyes. Because both the target and receptive fields are unchanged by rotation through 90°, the response is also unchanged. The top row of Fig 13 shows responses for disks at 2.5cm, close to the sensor disparity, and indeed the response as a function of time is very similar for the two directions of motion (though of course rotated as a function of (*x*_*c*_, *y*_*c*_) to reflect the direction of motion).

For other disparities, this is not so. For stimuli at greater simulated distances, the stimulus horizontal disparity becomes more mismatched with the sensor horizontal disparity. This reduces the response for both directions of motion, but the underlying reason is different depending on the target direction of motion, as we now explain.

For vertical motion, assuming that the target has no vertical disparity, the leading and trailing edges enter the excitatory region at the same time, but if the target disparity does not match the sensor disparity, they cannot both pass over its center. If the target has a suitable horizontal offset *x*_*c*_, it can pass over the optimal region in one eye, but in the other eye much of the target necessarily passes over the inhibitory region, causing a weak response. The best compromise is for *x*_*c*_ close to 0, when the target is only slightly misaligned in both eyes. Since the sensor receptive fields are symmetrically located about the midline, the sensor is tuned to stimuli on the midline, and so it responds most strongly to stimuli on the midline even when these do not have the preferred disparity. This is why the peaks are centred on *x*_*c*_ = 0 in Fig 13M–13X, the panels relating to vertical motion.

For a target moving horizontally (and with no vertical disparity), the target can pass over the central excitatory region in both eyes. However, if the target disparity does not match that of the sensor, the leading and trailing edges are offset relative to the receptive fields: compare Fig 12A–12C with Fig 14A–14C. This usually reduces the response.

However, as we noted in the previous section, for some combinations of target size and disparity, the leading edge can pass through the excitatory region in one eye at the same time as the trailing edge is passing through the excitatory region in the other eye, as in Fig 14B. The two mismatched edges thus combine to give a response in the disparity sensor comparable to the response in the top rows of Fig 13, where both leading edges crossed at the same time. This effect occurs when the difference between the target and sensor disparity is similar to the target diameter. For example, for a target at a simulated distance of 10cm, the target disparity is zero, and so the disparity relative to the sensor is the sensor screen disparity of 15.4°. For a target of diameter 17°, this is close enough for the leading and trailing edges to overlap substantially. This explains why a 17° diameter is the only size shown in Fig 13 where a target at 10cm still elicits a weak response (panel L).

### 3.3 Predicted striking behaviour as a function of vertical disparity

Rossel and colleagues [31] examined praying mantis striking behavior in response to stimuli with vertical (non-epipolar) disparity, introduced by means of prisms. They found that the sensitivity to horizontal disparity was unchanged, but that the strike rate declined with vertical disparity, reaching zero when the vertical disparity exceeded 15°. Strike rate depended on size, as already noted, but the dependence on vertical disparity was independent of size. The authors concluded that “the limit for vertical disparities is fixed, regardless of the size and microstructure of the target”.

**Fig 15:**
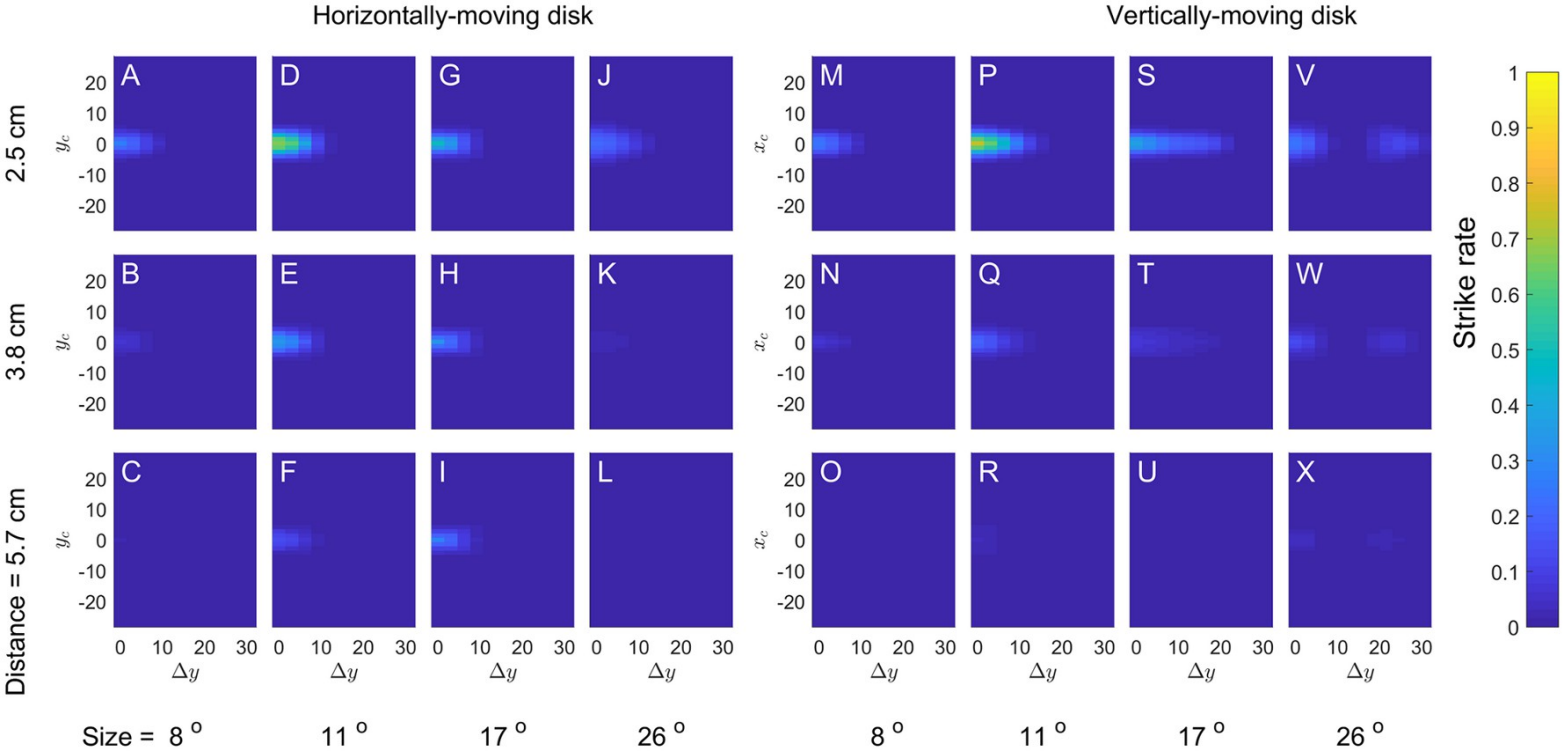
Predicted strike rate *M*_*model*_ (Eq 3) for disks moving horizontally (A-L) or vertically (M-X) in front of the model mantis, as a function of vertical disparity Δ*y* and offset perpendicular to the direction of motion (*y*_*c*_ for horizontally-moving targets and *x*_*c*_ for vertically-moving). For the horizontally-moving targets, the vertical location of the target in the two eyes is constant at *y*_*L*_ = *y*_*c*_ + Δ*y*/2 and *y*_*R*_ = *y*_*c*_ − Δ*y*/2. For vertically-moving targets, the mean *y*-location is of course a function of time, but the difference *y*_*L*_ − *y*_*R*_ is constant at Δ*y*. Different rows are for different disparities, thus simulating disks at different distances from the animal when Δ*y* = 0. Different columns are for different disk diameters.

We examined the response of our disparity sensor to stimuli with vertical disparity. Fig 15 shows the mean predicted strike rate, *M*_*model*_ (Eq 3), for targets moving horizontal and vertically, as a function of vertical disparity Δ*y* (shown on the horizontal axis). The vertical axis shows the offset of the target trajectory from the receptive field center, measured perpendicularly to the direction of motion. The different panels show results for targets of different sizes and simulated distances.

For all sizes and distances, the highest strike rate occurs for stimuli with zero vertical disparity and zero offset (i.e. passing directly over the receptive fields). In all cases, the strike rate falls to zero for vertical disparities of around 15°, corresponding to the extent of the excitatory region. For vertical disparities greater than this, it is impossible for the target to pass over the excitatory regions in both eyes simultaneously. However for large vertically moving disks that are close to the sensor’s preferred disparity, a low but non-zero strike probability is observed for very large vertical disparities. This is again the result of a “false match”, when the target’s trailing edge in one eye passes over the excitatory region at the same time as the leading edge in the other one.

**Fig 16:**
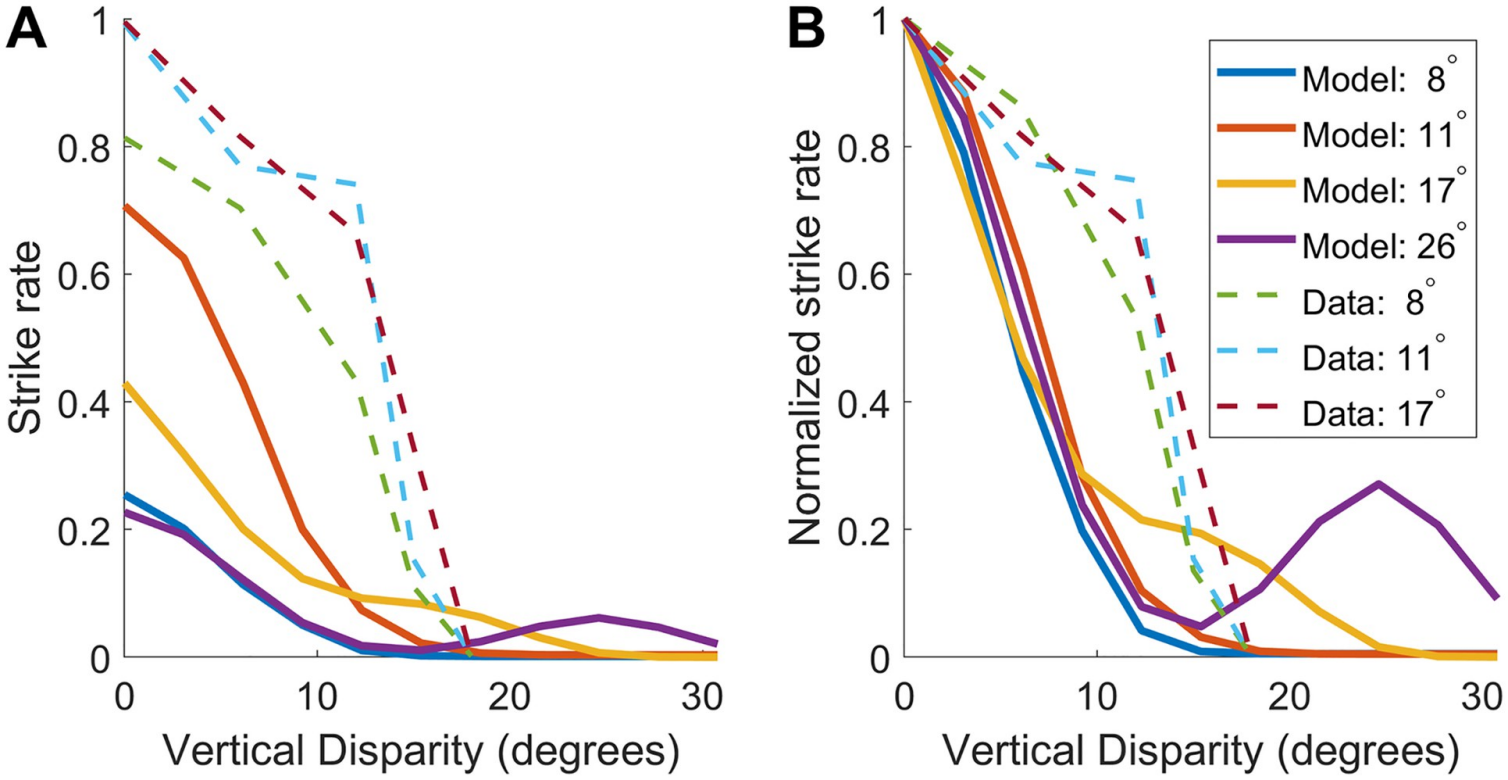
Sensitivity to vertical disparity and size, for our model (solid lines) and empirical data digitized from [31]. A: Solid lines show strike rate predicted by our model, averaged across horizontally and vertically travelling targets passing directly over the sensor. Dashed lines show strike rates observed by [31] for stationary “jiggling” targets. Colors indicate target size as shown in the legend. B: as A but the strike rate is normalized to be 1 for stimuli with zero vertical disparity.

Fig 16 plots the strike rates predicted by the model for zero offset as a function of vertical disparity (solid lines), and compares them with the empirical results of [31] (dashed lines).

### 3.4 Predicted striking behaviour for two targets presenting a ghost match

We have shown that our model predicts strikes to a single moving target at a distance of 2.5cm, as in Fig 10A. Rossel [30] examined mantis head saccades made to two distant targets, where the lines of sight to the more nasal target in each eye cross so as to form a “ghost match” at 2.5cm, as in Fig 10B. Rossel [30] showed that whereas mantids looked towards the midline in the single-target configuration of Fig 10A, they looked to left or right in the double-target configuration of Fig 10B, suggesting that they did not perceive an object at the location of the ghost match. In a similar geometry, humans likewise perceive two equidistant objects to left and right of the midline. In humans, this has been taken as evidence for cooperative interactions between disparity sensors, which act so as to favour matches with similar disparities; a form of global correspondence [2, 41].

We have examined mantis strikes made to stimuli offering ghost matches of this kind. We compared the four stimulus geometries shown across the top row of Fig 10A–10D. The baseline condition (A) presented a single virtual object at 2.5cm distance, simulated in the usual way by disparate images on a screen physically at 10cm. In condition (B), both images were presented to both eyes, thus depicting two objects at 10cm distance. In (C), we again presented two objects in each eye, but this time increased the separation between objects such that the retinal images could not be produced by any two physical objects. Note that all three conditions offer a ghost match at 2.5cm. Finally, as a control, we presented a single object at 10cm distance.

The results are shown in Fig 10I and 10J for small and large disks respectively. The rainbow-colored lines are for individual mantids, while the black dots represent the group mean ± standard error. As expected, the mantids strike enthusiastically at the single disk at 2.5cm (condition A). This sample of mantids actually strikes slightly more for the larger disk than the smaller, whereas the sample from [10] struck slightly more for the smaller disk (Fig 4A), suggesting a minor variation in size tuning between these two samples of mantids. Also as expected, the mantids make virtually no strikes to the single disk at 10cm, out of their catch range (condition D). Importantly, striking is also much less likely to the pairs of disks (conditions B, C), confirming Rossel’s conclusion that mantids do not perceive an object at the location of the ghost match.

We also tested our fitted model on these four stimulus geometries and sizes. The motion was the same as above, i.e. the targets moved either horizontally or vertically across the screen. The symbols in red in Fig 10I and 10J show model predictions for the two directions of motion, and for their average; Table 4 gives the figures for model and mantids.

The model’s agreement with data is excellent for the smaller disk, diameter 11.4°, close to the optimal size for the model Fig 11. For the larger disk, the model is incorrectly size-tuned and thus predicts fewer strikes than observed in every condition. This occurs because the model was not fitted to these data, but to a different sample of mantids with different size tuning and higher strike probability. However, the model captures the key observation, that the strike rate is far lower for the two-disk configurations, B and C. Thus like mantids, our model does not perceive the ghost match.

The reason for the model’s behavior is, of course, the inhibitory surround of the receptive fields. A single disk at the catch distance can excite both receptive fields and elicit strikes. However, a second disk, displaced by the amount needed to offer a ghost match, necessarily falls in the inhibitory surround and so suppresses the strikes which would otherwise have been elicited by the first disk.

## 4 Discussion

In this paper we set out to find out how well the praying mantis’ strike response to stimuli of different sizes and distances could be captured using a single disparity sensor. The binocular computation carried out by this model sensor is closely based on the stereo energy model. However, the early processing is different, incorporating lowpass spatial filtering, highpass temporal filtering and squaring. The model has 8 free parameters (Table 2): 5 which describe the size and weights of the monocular receptive field functions (*s*_*e*1_, *s*_*e*2_, *w*_*e*_1, *w*_*e*2_, *w*_*i*_), two describing binocular combination (*b* and *γ*) and one describing the preferred distance (*α*_*pref*_). We fitted these parameters to our empirical data [10] on strike rates for moving targets of a range of sizes and simulated distances. We also examined the predictions of the fitted model to stimuli with vertical disparity and to stimuli offering ghost matches, neither of which were used to fit model parameters.

We were able to find parameters that enabled this model sensor to capture the key features of interest (Fig 11): (1) strong tuning to disparity (strike rate reduced by a factor of 4 for a 5° change in disparity); (2) weak tuning to size (strike rate reduced by a factor of 4 for a 20° size in diameter. This confirms that our data can be modelled with a single sensor, tuned to both disparity and to size.

Our model is also qualitatively consistent with Rossel’s data on vertical disparity (Fig 16). Our data were not fitted to these results, which were from a different species with a larger size preference, so in our model the strike rate falls to zero at a smaller size than in Rossel’s data. However, the model shows the same phenomenon reported by Rossel: that the fall-off in strike rate with vertical disparity was independent of the stimulus size (Fig 16B), so that it is not the case that vertical disparity is tolerated so long as the stimuli in the two eyes overlap by a certain extent. Our model makes it clear that the limit on vertical disparity is set by the receptive field size, and thus by the animal’s preferred size rather than the size of the particular stimulus. The model thus proposes a neurophysiological explanation for Rossel’s finding of a fixed limit for vertical disparities.

Our model also explains how mantids are able to avoid being misled by “ghost matches” formed by pairs of identical stimuli Fig 10B and 10C. This does not require the global-correspondence mechanisms postulated to account for similar effects in human stereopsis; rather, it follows simply from the size-tuning. Stimuli which offer a ghost match necessarily stimulate the inhibitory surround as well as the excitatory centre of our disparity sensor, suppressing its response. Rossel [30] proposed that neurons with inhibitory surrounds could account for mantids’ immunity to ghost matches, and we have here confirmed this quantitatively. Our model gives a very good account to data from our lab on mantis strikes to ghost-match stimuli (Fig 10 and Table 4), even though this data-set was not used in fitting the model.

Our model produced three other surprising results, all due to false matches between the leading and trailing edges of the stimulus. First, for horizontally-moving stimuli, the model prefers larger angular sizes at further simulated distances. This effect is seen in our data [10], though since we used spiralling stimuli we cannot say whether it occurs only for horizontal trajectories. It was a puzzling result since it is the opposite of what would be required for physical size constancy We had not considered the possibility that false matches between leading and trailing edges could account for it. It is worth noting that Rossel did not find this effect [36], Fig 4, but he used stimuli with very different motion: targets moving slowly or “performing small oscillating movements”. As Rossel commented at the time [36], “Movement is also important and … it is possible that a different mode of target presentation would give a different result”.

Second, also for large horizontally-moving stimuli, the model predicts a low but non-zero strike rate for uncrossed disparities, which do not correspond to a single point in space. We saw a similar effect in our data but argued that it was not driven by disparity, so subtracted it before fitting. We now wonder if these strikes could have been driven by false matches in the mantis as they were in our model. Finally, the model predicts that vertically-moving stimuli, if much larger than the preferred size, can elicit strikes even when they have very large vertical disparity Fig 16. Since the only experiments with vertical disparity used stationary stimuli [31], we do not know whether such an effect would be observed empirically.

For uniform light stimuli like those used here, the leading edge represents a contrast increment while the trailing edge is a decrement. A false match between these two is only possible because our model includes a rectification step which abolishes the distinction between increments and decrements. Neurally, this would correspond to combining inputs without regard to whether they indicate increments and decrements, e.g. excitatory input from both L1 and L2 pathways [37]. An alternative would have been to postulate input only from the L2 pathway, which responds to moving dark edges. Mathematically, this would correspond to halfwave instead of full rectification, and would have the advantage of building in mantids’ preference for dark prey items [32], currently ignored in our model. However, this would have meant that the sensor would “see” only the leading edge of the target, and so would be insensitive to size parallel to the direction of motion, which is not what is observed. Our decision to make the sensor respond to both leading and trailing edges enabled us to achieve realistic size-tuning, but enables these false matches. It therefore becomes important to test these predictions of the model behaviorally, in order to understand whether mantis stereopsis can really be misled by false matches between the leading and trailing edges of a moving target, or whether mantids have a more sophisticated form of stereo correspondence which prevents this.

The above discussion raises the question of what aspects of mantis striking behaviour should be attributed to the properties of their disparity sensors, and what should be considered separate. The model discussed in this paper is sensitive to size and disparity, but—as just noted—not to contrast polarity, nor directly to other aspects of visual stimuli that influence strike rates, such as looming, speed of figure motion etc. We have argued previously [21] that mantis behaviour is most economically accounted for by postulating neural mechanisms for detecting prey which are distinct from those detecting binocular disparity: effectively, a “prey sensor” in addition to the disparity sensor modelled here. We proposed that striking requires the disparity sensor to be activated at the same time as, or within a limited time window after, the prey sensor, and that the strike rate reflects the activity in both systems (e.g. via a multiplicative interaction). We argued that this can explain why looming (an increase in angular size over time) increased strike rates independent of disparity [42]: looming activates the prey sensor but does not enhance the activity of the disparity sensor. It also explains why mantids can discriminate stereoscopic depth in stimuli with no figure motion, although they will not strike at these unless they first see stimuli with figure motion [21]: the figure motion activates the prey sensor, and if the disparity sensor is subsequently activated as well, strikes can occur. In principle, we could have attributed the size tuning also to properties of the prey sensor, but instead we chose to incorporate it within the disparity sensor directly. Two lines of evidence, one behavioural and one neurophysiological, motivated this decision. First, neurons sensitive to disparity have centre/surround receptive fields [22, 23], implying that they are tuned to size as well as to disparity. Second, the dependence of size tuning on disparity [10] suggests joint rather than separable mechanisms.

Our model disparity sensor is not tuned directly to speed or figure motion, but its temporal filtering makes it indirectly sensitive to target speed. This is because the speed affects the width of the leading and trailing edges across the retina. The squared output of the high-pass filter is above 14% of maximum for a time *τ* after the edge passes, so the width is *Vτ*. Slower stimuli thus offer less total excitation as they pass over the receptive field. Faster stimuli give wider edges, but once the stimulus gets fast enough that the leading and trailing edges merge, there is less total excitation. All in all, these effects lead to complicated interactions between speed, size and disparity tuning which are beyond the scope of this paper. We do not, however, believe that the current model is accurate in this regard. More accurate models of the spatiotemporal inputs to the disparity sensor will be required to ensure that model predictions remain accurate for stimuli moving at different speeds, and for stimuli more complicated than black disks (e.g. targets defined by luminance flicker or theta motion [13, 21]). This will require more detailed behavioral data, and would benefit greatly from further neurophysiological experiments elucidating the properties of the relevant neurons.

Finally, this model includes only a single disparity sensor. As noted in the Introduction, it is helpful to begin with the simplest possible model in order to understand what this is capable of, before elaborating it. In fact, neuroanatomical and physiological data suggests multiple disparity-tuned neurons, even within a given cell class [22], as well as multiple classes of disparity-tuned neuron whose role is unclear. Some may mediate other behavior, such as head saccades, which are also sensitive to stereoscopic information; others may implement top-down feedback, for example guiding visual attention in space. One simple extension of the present model would be to include a few different copies of this sensor, with receptive fields at different locations in the visual field. This would account for mantids’ ability to strike at targets over a range of the visual field around 20° in diameter. However, expanding the model to account for the full range of disparity tuning found in mantis brain must wait until more neurophysiological data is available regarding their response properties.
